# A Systems Level, Functional Genomics Analysis of Chronic Epilepsy

**DOI:** 10.1371/journal.pone.0020763

**Published:** 2011-06-14

**Authors:** Kellen D. Winden, Stanislav L. Karsten, Anatol Bragin, Lili C. Kudo, Lauren Gehman, Josephine Ruidera, Daniel H. Geschwind, Jerome Engel

**Affiliations:** 1 Interdepartmental Program for Neuroscience, University of California Los Angeles, Los Angeles, California, United States of America; 2 Program in Neurogenetics, University of California Los Angeles, Los Angeles, California, United States of America; 3 Department of Neurology, University of California Los Angeles, Los Angeles, California, United States of America; 4 Department of Neurobiology, University of California Los Angeles, Los Angeles, California, United States of America; 5 The Brain Research Institute, University of California Los Angeles, Los Angeles, California, United States of America; 6 Semel Institute for Neuroscience and Human Behavior, David Geffen School of Medicine, University of California Los Angeles, Los Angeles, California, United States of America; 7 Department of Human Genetics, University of California Los Angeles, Los Angeles, California, United States of America; 8 Department of Microbiology, Immunology, and Molecular Genetics, University of California Los Angeles, Los Angeles, California, United States of America; 9 NeuroIndx Inc., Signal Hill, California, United States of America; 10 Division of Neuroscience Research, Department of Neurology, Harbor-UCLA Medical Center, Torrance, California, United States of America; Consejo Superior de Investigaciones Cientificas - Instituto Cajal, Spain

## Abstract

Neither the molecular basis of the pathologic tendency of neuronal circuits to generate spontaneous seizures (epileptogenicity) nor anti-epileptogenic mechanisms that maintain a seizure-free state are well understood. Here, we performed transcriptomic analysis in the intrahippocampal kainate model of temporal lobe epilepsy in rats using both Agilent and Codelink microarray platforms to characterize the epileptic processes. The experimental design allowed subtraction of the confounding effects of the lesion, identification of expression changes associated with epileptogenicity, and genes upregulated by seizures with potential homeostatic anti-epileptogenic effects. Using differential expression analysis, we identified several hundred expression changes in chronic epilepsy, including candidate genes associated with epileptogenicity such as *Bdnf* and *Kcnj13*. To analyze these data from a systems perspective, we applied weighted gene co-expression network analysis (WGCNA) to identify groups of co-expressed genes (modules) and their central (hub) genes. One such module contained genes upregulated in the epileptogenic region, including multiple epileptogenicity candidate genes, and was found to be involved the protection of glial cells against oxidative stress, implicating glial oxidative stress in epileptogenicity. Another distinct module corresponded to the effects of chronic seizures and represented changes in neuronal synaptic vesicle trafficking. We found that the network structure and connectivity of one hub gene, *Sv2a*, showed significant changes between normal and epileptogenic tissue, becoming more highly connected in epileptic brain. Since *Sv2a* is a target of the antiepileptic levetiracetam, this module may be important in controlling seizure activity. Bioinformatic analysis of this module also revealed a potential mechanism for the observed transcriptional changes via generation of longer alternatively polyadenlyated transcripts through the upregulation of the RNA binding protein HuD. In summary, combining conventional statistical methods and network analysis allowed us to interpret the differentially regulated genes from a systems perspective, yielding new insight into several biological pathways underlying homeostatic anti-epileptogenic effects and epileptogenicity.

## Introduction

Epilepsy is a neurological condition with myriad etiologies that is characterized by the recurrence of spontaneous seizures [1]. One of the most common epilepsy sub-types is mesial temporal lobe epilepsy (MTLE), where the epileptogenic region is localized to the temporal lobe [1]. Pathologically, the hippocampus in humans and animals with MTLE reveals alterations in multiple cellular processes, including inflammation, cell death and synaptic reorganization [2]. Advances in genetics, electrophysiology, and molecular biology have already led to significant understanding of the types of genes and pathways that cause chronic seizures [3]. In addition, discovery of genes and loci associated with focal epilepsies in the temporal lobe promise to provide insight into the etiology of MTLE [4], [5]. Although the genetic contribution to MTLE seems negligible because MTLE is often precipitated insult to the brain [6], patients that develop MTLE are more likely to have a family history of epilepsy [7], [8]. Therefore, genetic factors may modify the development of epilepsy and generation of spontaneous seizures, and understanding the mechanisms by which temporal lobe lesions lead to chronic epilepsy will provide insight into the genetic basis of epileptogenicity and epilepsy susceptibility.

There are several models of MTLE that utilize the injection of a neurotoxin (i.e. kainic acid, pilocarpine) to induce a brief period of status epilepticus. After a latent period, these animals will go on to develop spontaneous, recurrent seizures [9]. Although these models accurately recapitulate many aspects of human MTLE, there are many interconnected factors that affect gene expression, including the effects associated with the lesion, seizures, and ability to generate spontaneous seizures (epileptogenicity). We focused on the effects associated with seizures and epileptogenicity because these changes are the most important for understanding epileptic processes. In addition, the alterations caused by seizures could provide insight into homeostatic, anti-epileptogenic mechanisms that must be overcome for seizures to occur.

The study of epilepsy at the molecular level has been greatly aided by the use of genome-wide expression microarrays, which provide expression information for all genes that are expressed within a biological sample. Several studies have used gene expression data to identify candidate genes involved in epileptogenesis, such as Cystatin C and Homer 1A [10], [11], but understanding the biological significance of these dysregulated genes has been more challenging. Recently, a study of the hippocampus throughout the development of epilepsy revealed differentially expressed genes involved in multiple processes, including cell death, complement activation, oxidative stress, glial activation, and GABA signaling [12]. However, this study was limited by the absence of a control for the epileptogenic hemisphere, resulting in the inability to differentiate between effects of epilepsy and the lesion. Additionally, the effects of chronic seizures that might reflect a homeostatic anti-epileptogenic response to seizures have rarely been studied or factored into these analyses.

Here, we used a model of MTLE where kainic acid (KA) is injected into one hippocampus to induce status epilepticus [13]. Approximately half of the animals developed spontaneous seizures, and we found that the epileptogenic region within the animals that developed epilepsy was localized to the lesioned hippocampus, consistent with previous studies [13]. Therefore, we reasoned that there must be underlying differences in the lesioned hippocampi of animals that developed seizures that explain their epileptogenicity. To identify these differences, we grossly examined these animals histologically and used whole genome microarrays to analyze gene expression differences. We focused on gene expression changes within the dentate gyrus because the pathological high frequency oscillations that have been found in the dentate are thought to demarcate the epileptogenic region within this animal model [13]. We used differential expression analysis to examine effects of epilepsy on gene expression. Initially, we separately identified the effects of the lesion and seizures and used these data to identify gene expression changes associated with epileptogenicity. We then used weighted gene co-expression network analysis (WGCNA) to distill a potentially long list of genes into a network structure that facilitates interpretation of these gene expression changes. Using both methods of analysis, we were able to identify and characterize genes and pathways that were associated with epileptogenicity and with the effects of chronic seizures.

## Materials and Methods

### Animals

The University of California, Los Angeles, Institutional Animal Care and Use Committee approved all procedures involving animals described in this study. The ARC protocol ID is #2000-153-32. Total of twenty-seven oubred male (250–300 g) Wistar rats were used in these studies. Adult male rats were given atropine (0.04 mg i.m.), anesthetized with isofluorane 2%, and unilaterally injected with kainic acid (KA; 0.2 µl; 2.0 µg/µl normal saline) in the right posterior CA3 area of hippocampus (AP = −5.6 mm, ML = 4.0 mm, DV = 7.0 mm. A 30ga. needle attached to a Hamilton 1.0 µl syringe was lowered into the injection site and after 5 minutes, one half of the volume was injected. After another 5 minutes, the needle was raised 0.5 mm and the remainder of the solution was injected. After 20 minutes, the needle was withdrawn. Beginning 2 months after injection, video monitoring was performed for all rats in order to detect spontaneous behavioral seizures. After eight months of video monitoring rats were divided for electrophysiological (n = 12), microarray (n = 10).

### Microelectrode implantation

Eight – twelve months after kainic acid injection rats selected for electrophysiological experiments were anesthetized with isofluorane, 2%. Pairs of tungsten wires (60 µm in diameter) with 0.5 mm vertical tip separation were placed in the right angular bundle to stimulate perforant path afferents to the hippocampus (AP = −7.0 mm from bregma, ML = 3.5 mm and DV = 2.5 mm from the surface of neocortex, Paxinos, Watson, 1997). Fixed recording microelectrodes also consisted of pairs of tungsten wires with 1.0 mm vertical tip separation. They were implanted bilaterally at symmetrical points in the dentate gyrus (DG) and CA1 region of anterior hippocampus (AP = −3.5, ML = 2.0, DV = 3.5–4.5), DG region of the posterior hippocampus (AP = −5.0, ML = 4.0 DV = 5.0); EC (AP = −8.0, ML = 5.0, DV = 7.0) and piriform cortex (AP = 2.2, ML = 4.0 DV = 6.5).

### Electrophysiological recordings

During *in vivo* recordings in freely moving rats, five 4-channel MOSFET input operational amplifiers mounted in the cable connector served to eliminate cable movement artifacts. Physiological data were recorded wide-band 0.1 Hz to 3.0 kHz and sampled at 10 kHz/channel (16 channels) with 12 bit precision using RC-Electronics (Santa Barbara, CA) software. Location of electrodes was determined on the basis of the shape of responses to perforant path stimulation. Recordings were performed during 1 month 5 days a week and 8–10 hours a day. After completion of electrophysiological experiments rats were given overdose of Nembutal and perfused for following histological verification of the location of the recorded electrodes and for neo-Timm staining for estimation of mossy fiber sprouting [14]. An image analysis program was used to quantify these data by counting silver deposit punctae and expressing these data as a density, by dividing by the total area [14].

### Data analysis

Recorded seizures were classified on the basis of electrophysiological onset pattern, signal averaging and power spectral analysis. For high frequency oscillations (HFOs) analysis raw records were band-pass filtered between 80–500 Hz, using a Butterworth filter with roll off 3 dB, which does not introduce any visible phase lag. Then HFOs were detected by DataPac (Mission Viejo, CA) software followed by averaging and power spectrogram analysis.

### Microdissections

Ten rats were taken for microarray studies: five with documented behavioral spontaneous seizures and five rats without spontaneous seizures. Rats were anesthetized with isofluorane, brains were quickly removed and sectioned by vibratome into 400 µm slices. Dentate gyri were removed under dissecting microscope and placed in −80C for further microarray experiments. Horizontal sections were taken approximately 6.2 mm below the bregma. The comparisons were performed between the area of dentate gyri (DG) adjacent to the KA lesion and non-lesioned contralateral side in each animal.

### RNA isolation and probe preparation

RNA extraction was performed using Trizol (Invitrogen). RNA concentration and quality were evaluated using Nanodrop ND-1000 spectrophotometer (NanoDrop) and Agilent 2100 Bioanalyser (Agilent). We used 100 ng of RNA as the initial starting template, which was labeled with Cy-3 or Cy-5 cytidine -5′-triphosphate (CTP) using the low input fluorescent linear amplification kit (Agilent). The labeled cRNA concentration was verified (Nanodrop) and RNA quality was checked on an Agilent Bioanalyzer (Agilent).

### Microarray platforms

Two commercial oligonucleotide microarray platforms, the Rat Microarray carrying 20,500 probes representing 15,703 unique GeneBank IDs (Agilent Technologies, CA) and the CodeLink Rat Whole Genome array with 33,664 probes representing 24,329 unique GeneBank IDs (GE Healthcare) were used to identify gene expression changes. Both platforms contained a total of over 54,164 distinct oligonucletide probes. Due to partial overlap in the gene lists of Agilent and CodeLink microarray platforms, the total list of probes corresponded to 29,080 unique GeneBank sequences that represented 14,439 unique genes with known full-length cDNA sequence (including RIKEN genes) and additional 14,641 expressed sequence tags (ESTs). 9,879 genes were represented by more then one probe, which typically recognized different parts of the transcript. For the Agilent microarrays, the right dentate gyrus from one animal was labeled with Cy-5, and the left dentate gyrus from the same animal was labeled with Cy-3. These were then hybridized to the same slide. The dyes were then swapped, and the same samples were hybridized to another slide. For the Codelink microarrays, all samples were labeled and hybridized in a random order to two chips, each containing twelve arrays. As a result, each individual sample was hybridized to at least three different microarrays (2 Agilent & 1+ Codelink). Microarray hybridization and scanning was performed according to manufacturers protocols. We have deposited the raw data at GEO under accession numbers GSE27015 (Agilent) and GSE27166 (Codelink), and we can confirm all details are MIAME compliant.

### Microarray data analysis

We imported the raw data from the Agilent and Codelink microarrays into R (http://www.r-project.org/). We used available libraries from Bioconductor (http://www.bioconductor.org/) to analyze the one-color and two-color microarray designs. For the two-color Agilent data, we used the linear models package (LIMMA). After the removal of flagged data, we used the default method for background subtraction and used quantile normalization to normalize values between arrays. We used the empirical Bayesian algorithm to determine differential expression and fold changes between different conditions. For the one-color Codelink data, we removed all of the flagged data, as well as all of the control probes. We used the quantile method to normalize the data. A Bayesian ANOVA was used to compare different conditions and determine differential expression.

In order to determine differential expression, we used the corrected p-value threshold (FDR)<0.10 and fold change >1.2 for both platforms. Using BLAST, we then mapped probes between the two platforms to determine those that targeted the same exon of the same gene. If a differentially expressed probe targeted the same exon as a non-differentially expressed probe on the other platform, then we required that the two probes be moderately correlated (r>0.5) for that gene to be called differentially expressed. All probes that targeted an exon uniquely and met the differential expression criteria were called differentially expressed.

We performed four major analyses to identify differentially expressed genes. 1) Differential expression between the injected and non-injected hippocampus in animals without seizures. 2) Differential expression between the injected and non-injected hippocampus in animals with seizures. 3) Differential expression within the injected hippocampi between animals with seizures and animals without seizures. 4) Differential expression within the non-injected hippocampi between animals with seizures and animals without seizures.

### DAVID gene ontology analysis

The genes that were identified as differentially expressed were used for gene ontology and network analyses. We used DAVID (http://david.abcc.ncifcrf.gov/) to identify over-represented functional categories within each group of differentially expressed genes [15]. Annotations were imported into DAVID, and all categories with an uncorrected EASE score of less than 0.05 were kept as significant.

### Network analysis

To identify the gene expression patterns in the dataset in an unbiased manner, we performed weighted gene co-expression network analysis (WGCNA) [16], [17], [18]. The data collected on the Agilent platform was used for WGCNA because there were more arrays. Initially, the data was normalized using quantile normalization and outlier arrays were removed. We selected genes based on high coefficient of variation, and pairwise Pearson correlations were calculated between all of these genes. We then raised these correlations to a power to approximate scale free topology within the network. From these scaled correlations, we calculated the topological overlap (TO) between all genes, which summarizes the degree of shared connections between two genes. Genes were then clustered based on their TO and then visualized in a dendrogram. Branches of the dendrogram are then isolated using a dynamic tree cutting algorithm [19], which correspond to groups of co-expressed genes. We summarized gene expression within a module using the first principal component of gene expression, and we term this value the “module eigengene” (ME) or the most representative expression pattern within the group of genes. For each gene, we determine module eigengene based connectivity (*k*_ME_) by calculating the absolute value of the Pearson correlation between the expression of the gene and the ME. The *k*_ME_ of a gene is related to its module centrality and importance to organization of the rest of the module [17].

### Cell type analysis

To determine which cell types that genes in a module were associated with, we used two published resources. The first was a database of gene expression from purified mouse neurons, astrocytes, and oligodendrocytes [20], and we imported the raw microarray data into R and normalized the data using the *affy* package, using optimal parameters [21]. We matched genes within a specific module to corresponding Affymetrix probes based on gene symbols and examined the expression of these genes visually. The second resource identified co-expressed groups of genes that corresponded to specific human neural cell types [17]. We identified the genes from our seizure-related modules in this cell type analysis based on gene symbol, and we calculated the average *k*_ME_ for those genes within each of the conserved modules. The average *k*_ME_ from our genes of interest was compared to the average *k*_ME_ for a random group of genes that was the same size, and we calculated a Z-score for each of our epilepsy-related modules within all of the consensus modules in the human cortical transcriptome.

### Quantitative RT-PCR

To validate the expression changes, we used qRT-PCR as previously described [22]. Briefly, RNA was converted to cDNA using the First Strand Synthesis Kit (Invitrogen). Gene specific primers were designed to amplify ∼100 bp regions of target genes (Table S1). Amplification was detected using Sybr Green (Bio-Rad) in a Light Cycler 3900 (ABI). For each gene in each experiment, a sample without reverse transcriptase was included to demonstrate specificity and lack of DNA contamination. Relative quantification was used to determine the abundance of each gene. The delta delta Ct method was used to calculate the fold change of each gene relative to the loading control beta actin.

### In situ hybridization

Gene specific probes were designed to be ∼500 bp and directed against same regions that the microarray probes targeted (Table S2). *In situ* hybridization was performed as previously described [23]. Briefly, epileptic and non-epileptic rats were sacrificed, the brains were dissected out, and they were flash frozen on dry ice. Coronal sections were cut using a cryostat. Radioloabeled nucleotide probes were hybridized onto the sections, and they were allowed to develop for eight days.

## Results

### Phenotypic characterization of seizures

**Figure 1:**
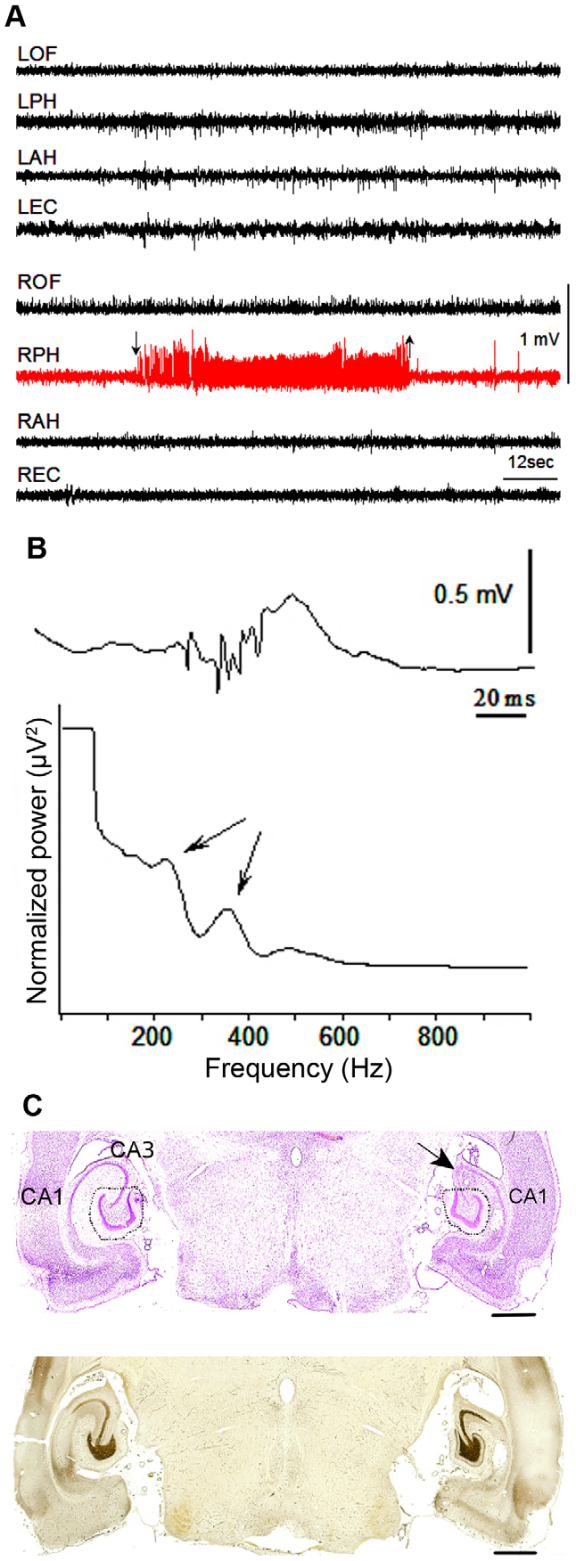
Characterization of seizure model. Using electrophysiological and histological methods, we characterized a subset of animals that received KA injection. a) Sample *in vivo* electrophysiology recording, demonstrating a spontaneous seizure in the hippocampus of the lesion side. In total, eleven seizures were recorded in the group of epileptic animals. Eight seizures (73%) started in the right posterior hippocampus in the area adjacent to the KA lesion and did not spread to other brain areas, and three seizures (27%) started simultaneously in right and left hippocampi. Seizures in this animal model have been previously characterized [13], [77]. Abbreviations: R/LOF – right/left orbital frontal, R/LPH – right/left posterior hippocampus, R/LAH – right/left anterior hippocampus, R/LEC – right/left entorhinal cortex. b) Example of pHFOs recorded from the dentate gyrus of animals with chronic epilepsy, where the fast ripples occur simultaneously with an interictal spike. Averaged power spectra of fast ripples recorded from the dentate gyrus, where the arrows denote the presence of physiological ripples at ∼200 Hz and pHFOs at ∼400 Hz. c) Representative, horizontal sections from one epileptic animal stained by cresyl violet and Timm method eight months after KA injection. In the right hippocampus, the CA3 area is absent due to the KA lesion (indicated by arrow). Dotted lines indicate areas that were dissected for microarray experiments. Timm staining demonstrates moderate mossy fiber sprouting is on the lesion side. Scale bar = 1 mm.

We examined an animal model of focal epilepsy in which KA is injected into the hippocampus of one hemisphere. Out of the twelve animals used for detailed electrophysiological and behavioral characterization of seizures, we observed that five (42%) animals had clear behavioral seizures during the monitoring session and seven (68%) did not have clinically observable seizures. Within the seven animals that did not display behavioral seizures during video monitoring, electrographic seizure activity was recorded in two animals. This is consistent with the fact that some animals with KA treatment demonstrate electrographic ictal events without evident behavioral seizures. A majority of the recorded seizures began in the posterior hippocampus of the lesioned hemisphere (Figure 1a), which suggested that the epileptogenic region was commonly localized to the lesioned hemisphere. These data are supported by the presence of pathological high frequency oscillations during interictal periods in the right posterior hippocampus in 3 of 5 animals with seizures and none of the animals without seizures (Figure 1b). Pathological high frequency oscillations (250–600 Hz) have been found within epileptogenic areas of the dentate gyrus [24]. Therefore, these data demonstrate that our intrahippocampal injection of KA models focal epileptogenic processes in the lesioned hemisphere, as has been shown previously [13].

To understand the pathological processes initiated by KA that are involved in the ability to generate seizure activity (epileptogenicity), we undertook detailed examinations of multiple animals with and without seizures on both morphological and histological levels. The study design allowed us to compare animals with and without seizures, despite the fact that all animals had experienced an initial neurotoxic lesion. Cell death is thought to be important in the development of epilepsy [2], and therefore, we examined the gross histology of the lesioned hemisphere of multiple animals to determine whether animals that developed epilepsy demonstrated greater degrees of cell death. In both animals with and without seizures, the area of the dentate gyrus on the lesion side was smaller than the non-lesioned side (Figure 1c), but there was no obvious difference between the animals with and without spontaneous seizures. We then examined mossy fiber sprouting because it has also been thought to be important for epileptogenic processes [2]. However, Timm staining revealed that there was no significant difference in mossy fiber sprouting between animals with and without seizures (with seizures 1.41±0.27SD; without seizures 1.16±0.10SD) (Figure 1c). Therefore, these data demonstrate that the processes leading to the epileptogenicity of the lesioned hemisphere in this animal model cannot be explained by differences in cell death or mossy fiber sprouting, and we reasoned that changes associated with the development of epilepsy might be reflected on the molecular level.

### Genome-wide analysis of differential gene expression in seizures and lesions

**Figure 2:**
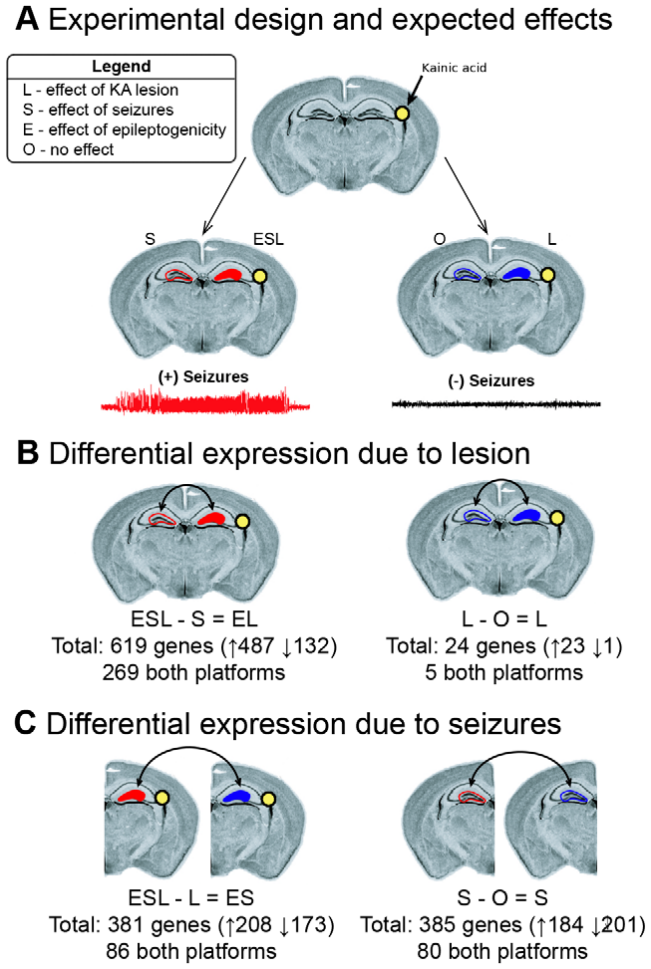
Experimental design and differential expression analysis. Animals were injected with KA as described in the methods, and gene expression between different samples was compared to identify the effects of seizures and neurotoxic lesion. a) All animals were injected with KA into the right hippocampus but only half of the animals developed chronic epilepsy. Expression in both hippocampi from five animals that developed chronic seizures and five that did not develop chronic seizures was investigated using both the Agilent and Codelink microarrays. The expected effects of interest are illustrated, including 1) the effect of the lesion (L), 2) the effects of seizures (S), and 3) the effects associated with epileptogenicity (E). b) We used two differential expression analyses to identify gene expression changes caused by the lesion. We found 619 differentially expressed genes between the lesioned and non-lesioned hippocampi in animals with seizures (ESL-S = EL) and 24 differentially expressed genes between the lesioned and non-lesioned hippocampi of animals without seizures (L-O = L). c) We then used two differential expression analyses to identify gene expression changes caused by seizures in the lesioned and non-lesioned hippocampi. We identified 381 differentially expressed genes between animals with and without seizures in the lesioned hippocampus (ESL-L) and 385 differentially expressed genes between animals with and without seizures in the non-lesioned hippocampus (S-O).

Ten additional animals were used for analysis of gene expression in chronic epilepsy. The dentate gyri of five animals with documented chronic behavioral seizures were isolated by microdissection, as well as equivalent regions in five animals that did not develop epilepsy (Methods; Figure 2a; Table S3). We took a conservative approach in our categorization of animals and separated animals into groups based on overt clinical seizures. Although it is possible that some animals without obvious seizures may have had sub-clinical seizure activity, thus decreasing our power to identify changes associated with epilepsy, our conservative approach would enable us to have more confidence in any expression changes observed. Left and right dentate gyri were processed separately to enable us to distinguish gene expression changes between the lesioned side (right) and the non-lesioned side (left) in animals with and without seizures. In addition, we used two independent microarray platforms (Agilent and Codelink) to expand our search for gene expression changes and allow for cross validation of expression changes with an independent method. In total, 64 microarrays across both array platforms were analyzed, profiling the left and right dentate gyri separately from animals with (n = 5) and without seizures (n = 5).

We identified differentially expressed genes from both microarray platforms independently (see Methods). For each comparison (see below), we used a false discovery rate (FDR)<0.10 and a fold change >1.2 as criteria to determine differential expression. We were confident in differentially expressed genes at these liberal FDR and fold change thresholds because we will use several comparisons between platforms and conditions to help eliminate false positives. Because different probes that are associated with the same gene may correspond to different exons and measure different splice variants, we carefully compared all differentially expressed probes across the two platforms for their specific location within their target genes (see Methods). To perform this comparison, we used all differentially expressed probes and identified the corresponding probes on the other platform that targeted the same gene. In the simplest case, a probe uniquely targeted a gene and there was no corresponding probe on the other array, and we considered the gene differentially expressed (n = 519). Within the group of genes that were targeted by a probe from each platform, we determined whether or not the two probes targeted the same exon. If a differentially expressed probe did not have a corresponding probe targeting the same exon, then the gene was considered differentially expressed because the probe may represent a differentially regulated splice variant (n = 450). However, if the differentially expressed probe and corresponding probes from the other platform targeted the same exon, we compared the probes using a Pearson correlation, and if the probes were moderately correlated (r>0.5), we considered the gene to be differentially expressed (n = 440). We used this conservative correction based on the correlation to limit false positives caused by noisy or non-specific probes.

### Expected effects on gene expression

The experimental design permitted us to separate three main effects on gene expression of major interest: 1) the effect of the KA-induced lesion that is unrelated to epilepsy (lesion; L), 2) the effect of chronic seizures on the brain (seizure; S), and 3) gene expression changes associated with the ability to generate spontaneous seizures (epileptogenicity; E). We reasoned that the effect of neuronal injury due to KA injection (L) would be seen in the lesioned hippocampus of both animals with and without seizures (Figure 2a). In addition, we expected that the effects of chronic seizures (S) would be seen in both the lesioned and unlesioned hippocampi of the animals with seizures (Figure 2a). Finally, we reasoned that the effects associated with epileptogenicity (E) would only be seen in the lesioned hippocampus of animals with seizures (Figure 2a). We used these classifications to filter the observed gene expression effects in subsequent analyses of differential expression.

### Effects of the lesion

**Figure 3:**
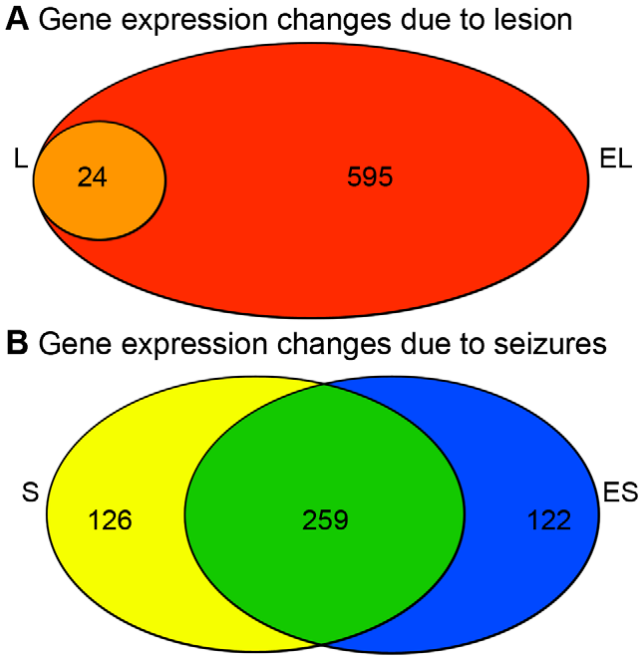
Comparisons of differentially expressed genes. The overlap between the two lists of differentially expressed genes due to the lesion and the overlap between the two lists of differentially expressed genes due to seizures are shown in the Venn diagrams. a) We compared the genes differentially expressed due to lesion in animals without seizures (L-24 genes) and with seizures (EL-619 genes) and found that all 24 genes that were differentially expressed due to lesion in animals without seizures were also differentially expression in animals with seizures, which is a significant overlap (orange; p = 2.06e-120). In addition, we found that 595 genes that were differentially expressed due to the lesion animals with seizures did not overlap with the core effect of the lesion (EL-L; red). b) We then compared the genes differentially expressed due to seizure in the lesioned (ES-381 genes) and non-lesioned hippocampi (S-385 genes) and found a large and significant overlap (green; n = 259 genes; p<1e-200). We also found that 122 genes that were differentially expressed due to seizures in the lesioned hippocampus did not overlap with the core effect of seizures (ES-S; blue).

To identify the specific effect of the lesion, we identified genes that were differentially expressed between the lesioned and non-lesioned hippocampi of animals without seizures (L-O = L). This analysis led to the identification of 24 differentially expressed genes that were specifically associated with the long-term effects of the lesion and independent of any epileptic processes because these animals did not have seizures (Figure 2b; Table S4). Interestingly, when we performed the same analysis on animals with seizures (ESL-S = EL), we identified many more significant gene expression changes associated with the lesion side (n = 619 genes; Figure 2b; Table S5). By comparing the two lists of differentially expressed genes due to the lesion, we found that all 24 of the genes that were differentially expressed due to the lesion in animals without seizures were also differentially expressed in animals with seizures (Figure 3a). Because histological comparisons between animals with and without seizures showed similarly-sized lesions and similar cytological effects, these data suggest not surprisingly that there is a substantial interaction between the initial pharmacologic lesion and chronic seizures, which may be related to epileptogenicity.

Gene ontology (GO) tools, such as DAVID, annotate the functional aspects of genes based on published literature and can identify any enrichment in these functional categories among a list of genes. We used GO analysis to begin to characterize the biological functions of the 24 genes specifically associated with the response to the lesion, and despite the fact that this list is small and therefore relatively underpowered for such analyses, this group was significantly enriched for genes involved in cell death (p = 7.49e-3) and the acute inflammatory response (p = 1.45e-2) (Table 1). In addition, *Gfap* and *Vim* were differentially expressed, and they are known to be present in reactive astrocytes [25]. Therefore, these data are consistent with the expected toxic effects of KA on the hippocampus.

**Table 1:** Functional annotation of genes that were differentially expressed due to the effects of the lesion.

Category	Term	P-value
*Biological process*	Cell death	0.0074
	Acute inflammatory response	0.014
	Actin filament depolymerization	0.028
	Metal ion transport	0.034
*Cellular component*	Cytoskeleton	0.0021
	Intrinsic to plasma membrane	0.031
*Molecular function*	Metal ion transmembrane transporter	0.007

GO analysis of the 24 differentially expressed genes that were changed solely due to the injection of KA. Enriched functional categories associated with the lesion were mostly related to cell death and inflammation, which is consistent with the expected effects of the KA injection.

### Effects of seizures

To identify the specific effects on gene expression due to chronic seizures alone, we identified differentially expressed genes between the non-lesioned hippocampi of animals with and without seizures (S-O = S). This analysis led to the identification of 385 differentially expressed genes that were solely related to the effects of chronic seizures and unrelated to the effects of the lesion (Figure 2c; Table S6). Because seizures were associated with such a large effect on gene expression, we tested whether the response to chronic seizures was similar in both hippocampi. Therefore, we performed the parallel analysis of the lesioned hippocampus by identifying genes that were differentially expressed between the lesioned hippocampi of animals with and without seizures (ESL-L = ES). This analysis identified 381 differentially expressed genes (Figure 2c; Table S7), which we hypothesized to be related to both chronic seizures and epileptogenicity. We then compared these two groups of differentially expressed genes due to seizures in the non-lesioned (Figure 3b; yellow) and lesioned (Figure 3b; blue) hippocampi, and we found that approximately 70% of the genes differentially regulated by seizures were shared between the two groups (Figure 3b; green; n = 259; p<1e-200). This remarkable and highly significant overlap represents the substantial impact that chronic seizures have on brain gene expression and that this effect is consistent, and independent of the lesion.

To investigate the functional consequences of the effect of chronic seizures at the molecular level, we used GO analysis on the differentially expressed genes due to seizures in both the lesioned and non-lesioned hippocampi. These groups of genes shared many enriched biological processes, including synaptic transmission (p = 5.91e-6 & p = 3.25e-6), secretion by cell (p = 9.15e-5 &p = 3.78e-4), and regulation of neurotransmitter levels (p = 6.47e-4 & p = 2.14e-4) (Table 2). Thus, chronic seizures have a large and consistent effect on gene expression and these changes suggest alterations in neuromodulation and neurotransmission, which is likely a result of plasticity induced by chronic seizures. However, based on these data alone, we cannot determine whether these changes in neurotransmission affect seizure activity in a causal or compensatory manner.

**Table 2:** Functional annotation of genes that were differentially expressed due to the effects of seizures.

Category	Term	P-value
*Biological Process*	Synaptic transmission	6.4e-5
	Secretory pathway	7.1e-4
	Regulation of neurotransmitter levels	0.0029
	Dopamine metabolic process	0.0043
	Protein transport	0.005
*Cellular component*	Coated vesicle	4.2e-4
	Cytoskeleton	0.015
	Axon	0.023
*Molecular function*	Transcription coactivator activity	0.019

GO analysis of the 259 differentially expressed genes that were changed due to the effect of chronic seizures on the brain. Multiple functional categories associated with transport and synaptic functions were enriched within this list of genes, suggesting that chronic seizures alter neuronal function or transmission.

### Effects associated with epileptogenicity

**Figure 4:**
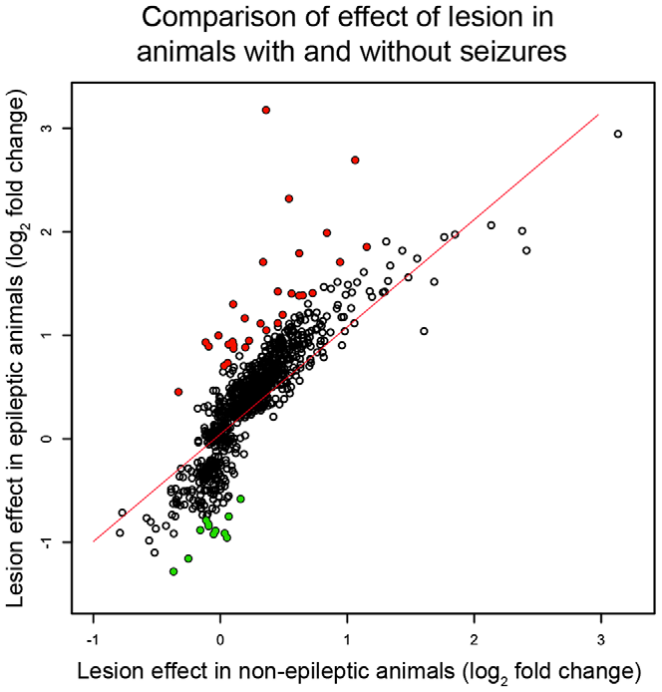
Most differentially expressed genes within the epileptogenic region were related to the effects of the lesion. We examined the effect of the lesion on the genes that were specifically differentially expressed within the epileptogenic region to determine whether they were plausible candidate genes to be associated with epileptogenicity. The scatter plot depicts the comparison of the lesion-induced fold changes between the animals with and without seizures for all of the genes that were specifically differentially expressed in the epileptogenic region. The x-axis represents the mean fold changes between the lesioned and non-lesioned hippocampi in animals without seizures and the y-axis represents the means fold changes between the lesioned and non-lesioned hippocampi in animals with seizures. All of the fold changes were transformed using log_2_. We found that there was a high correlation between the expression changes in animals with seizures and animals without seizures (r = 0.84), demonstrating many of these expression changes were related to the lesion. To remove this confounding factor, we used a linear regression to define the relationship between the effect of the lesion in animals with seizures and the effect in animals without seizures (red line). We then removed all genes that were within two standard deviations of this line (empty points), and after subtracting these genes, we identified 40 genes whose gene expression changes were not related to the lesion or seizures (12 down-regulated – green; 28 upregulated – red).

We hypothesized that candidate genes and pathways associated with epileptogenicity would be specifically differentially expressed within the lesioned hippocampus of animals with seizures. Two of the previous analyses identified differentially expressed genes that corresponded to epileptogenicity. The first group represented the 619 genes differentially expressed between the lesioned and non-lesioned hippocampi in animals with seizures (Figure 2b), which after subtracting the core effect of the lesion (n = 24 genes), amounted to 595 genes (Red; Figure 3a). The second group represented 381 differentially expressed genes in lesioned hippocampus due to seizures (Figure 2c), and we subtracted out the effect of seizures (n = 259 genes) and were left with 122 differentially expressed genes (Figure 3b, blue). These 595 and 122 differentially expressed genes represent expression changes within the epileptogenic region, but we found that many of these genes demonstrated similar expression changes between the lesioned hippocampus in animals with seizures and the lesioned hippocampus of animals without seizures (Figure 4; r = 0.84). These data suggest that the expression trend for many of these genes was related to the effects of the lesion, although the expression changes did not reach our statistical threshold for differential expression in the animals without seizures. Therefore, we used a linear regression to define the relationship between the effects of the lesion in animals with seizures and the effects of the lesion in animals without seizures, and we removed all of the genes that were within two standard deviations of this line. There were 40 remaining genes whose expression changes cannot be accounted for by either the effect of the lesion or the effect of seizures, including *Kcnj13*, an inwardly rectifying potassium channel, and *Ptgds*, which is involved in prostaglandin metabolism (Figure 4; Table 3). Therefore, these differentially expressed genes are candidate genes associated with epileptogenicity. Interestingly, GO analysis demonstrated that this list of genes is involved in neurotransmitter secretion (p = 1.1e-3) and neuronal differentiation (p = 3.3e-2). In addition, multiple neuropeptides (*Bdnf*, *Tac2*, and *Cart*) were present in the list and dramatically down-regulated in the epileptogenic region, as well as survival genes, such as bcl-2 related protein *Bok*. These data suggest that neuronal survival or function may be compromised within the lesioned hippocampus, increasing the epileptogenicity of that region.

**Table 3:** Candidate genes associated with epileptogenicity.

Function	Symbol	Gene title	Change
*Signaling*	Hcrt	Hypocretin	↑
	Ccl4	Small inducible cytokine a4	↑
	Kcnj13	Potassium inwardly rectifying channel, subfamily J, member 13	↑
	Carhsp1	Calcium regulated heat stable protein 1	↑
	Rasd2	RasD family, member 2	↑
	Ptgds	Prostaglandin D2 synthase	↑
	Ren1	Renin 1	↑
	Penk	Preproenkephalin	↑
	Bdnf	Brain derived neurotrophic factor	↓
	Unc13c	Unc-13 homolog	↓
	Grm4	Glutamate receptor, metabotropic 4	↓
	Cart	Cocaine and amphetamine regulated transcript	↓
	Olfm1	Olfactomedin 1	↓
	Prg1	Plasticity regulated gene 1	↓
	Slc30a3	Zinc transporter – Znt3	↓
	Tac2	Tachykinin 2	↓
	Comt	Catechol-o-methyltransferase	↓
*Differentiation*	Mcam	Melanoma cell adhesion molecule	↑
	Txnip	Upregulated by 1,25 dihydroxyvitamin D3	↑
	Fos	FBJ murine osteosarcoma viral oncogene homolog	↑
	Igfbp5	Insulin-like growth factor binding protein 5	↑
	Nnat	Neuronatin	↑
	Bok	Bcl-2-related ovarian killer protein	↓
	Nr4a3	Neuron-derived orphan receptor	↓
	Rgd1359691	Hypothetical protein Loc287543	↓
*Metabolism*	Loc311548	Similar to Riken cDNA 4930509O20	↑
	Eno2	Enolase 2, gamma	↑
	Ca3	Carbonic anhydrase 3	↑
	Cyb5r2	Similar to cytochrome B5 reductase	↑
	Cyp2d1	Cytochrome p450, family 2, subfamily D, polypeptide 9	↑
*Other*	Cdh19	Cadherin 19, type 2	↑
	Tmem10	Transmembrane protein 10	↑
	Samd14	Similar to cDNA sequence BC034054	↑
	Dnah1	Dynein, axonemal, heavy polypeptide 1	↑
	Rgd1310892	Similar to axoneme central apparatus protein	↑
	Cd86	Cd86 antigen	↑
	Samsn1	SAM domain, SH3 domain, and nuclear localization signals 1	↑
	Mucdhl	Mucin and cadherin like	↑
	Bcas1	Breast carcinoma amplified sequence 1	↑

The 40 genes that are specifically differentially expressed within the epileptogenic region and whose expression changes are not due to the effects of the lesion or seizures. These genes are grouped based on function from their associated GO categories. The arrows in the last column denote the direction of the expression changes within the epileptogenic region. There were twelve genes that were significantly down-regulated, and these genes were either associated with signaling or differentiation. The twenty-eight genes that were significantly upregulated within the epileptogenic region were spread out among all functional categories.

### Gene co-expression network analysis reveals higher-level organization of transcription

Standard analysis of differential expression allowed us to identify some of the pathological processes and dysregulated genes that are present in this animal model of epilepsy. But, while we identified strong, consistent effects associated with the lesion and seizures, the effects associated with epileptogenicity were subtler. We have recently demonstrated the power of systems biology approaches that utilize network methods to organize gene expression data through the use of weighted gene co-expression network analysis (WGCNA) [17], [18], [22], [26]. WGCNA is an unbiased and unsupervised method of gene expression analysis, which means that it does not depend on arbitrary differential expression thresholds. Importantly, WGCNA is independent of any characteristics associated with the sample, and therefore, any over-simplification of the seizure phenotypes that we assigned earlier will not confound this analysis. We used this method to identify groups of co-expressed genes or modules corresponding to major functional elements that contribute to gene expression changes in our epilepsy model, as well as hub genes that are central to the underlying biological processes. In addition, we have previously shown that WGCNA can identify modules that correspond to genes expressed in a specific cell types from complex tissue, such as human brain, permitting *in silico* tissue dissection [17]. Therefore, understanding the modular organization of gene expression would allow us to explore whether pathways within specific cell types were dysregulated due to chronic seizures, without the need for elaborate tissue dissection [17]. Using these methods, we were able to place observed gene expression changes into a systems context that was then related to the underlying biology.

WGCNA grouped the 11,000 expressed genes into forty co-expression groups or modules (see Methods; Figure S1). We summarized the gene expression within each module using the first principle component, which we term the module eigengene (ME). Within each of these modules, we calculated the Pearson correlation between the expression of each gene and the ME to determine ME-based connectivity (*k*_ME_), which is a measure of module centrality. We have previously shown that these central or hub genes are important for module function and organization [18], and we used this organization to annotate genes and rapidly identify the most important expression changes. To assess whether these co-expression relationships corresponded to changes due to seizures, injection, or other aspects of biological variability, we correlated each ME with the three major effects that were of interest. We identified two modules that were significantly related (r>0.5, p<0.005) to the effects associated with epileptogenicity and ten modules that were related (r>0.5, p<0.005) to the effect of chronic seizures.

### Co-expression module suggests key role for oxidative stress in epileptogenicity

**Figure 5:**
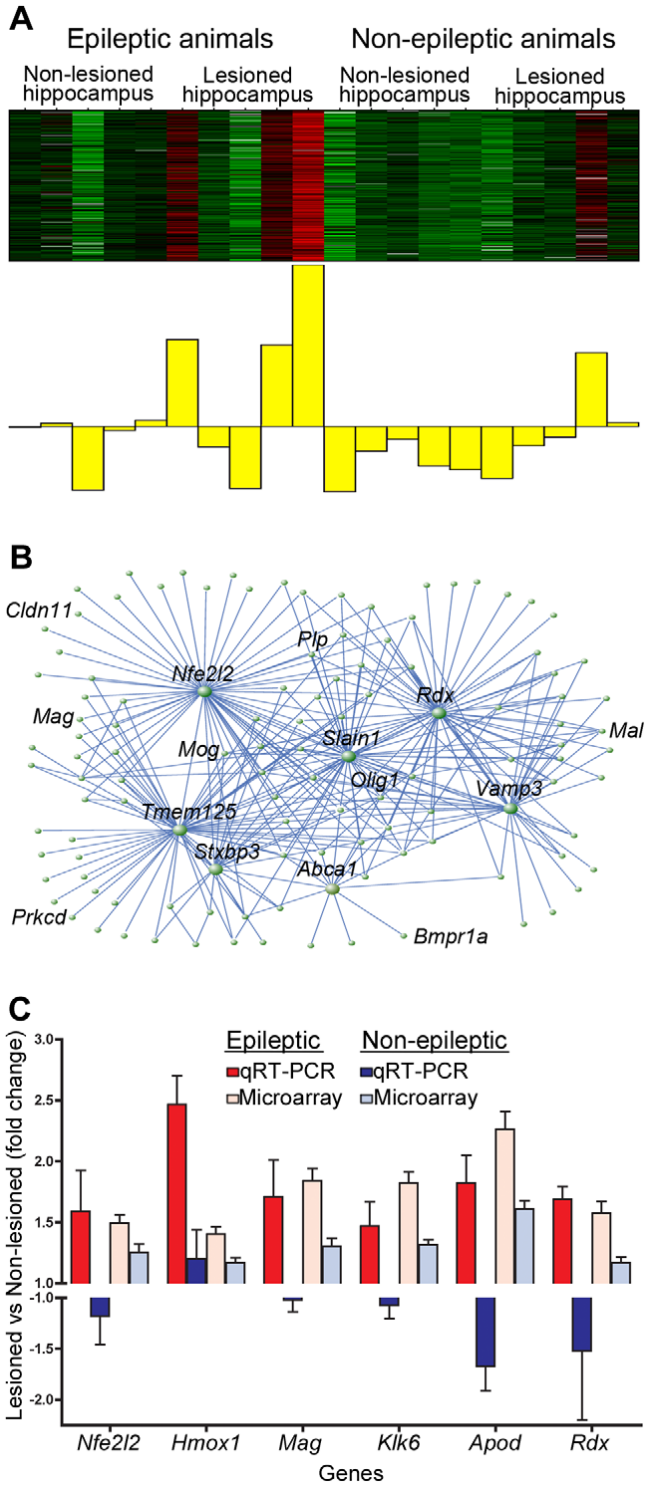
Yellow co-expression module contains genes upregulated in the epileptogenic region. Using WGCNA, we identified the yellow co-expression module that contained genes that were specifically upregulated in the lesioned hippocampus of animals with seizures. a) Heatmap of expression within the yellow module, where each sample represents a column and genes are displayed in the rows. Expression is scaled for each gene, where red denotes that the gene is highly expressed in that sample and green denotes low expression. Below the heatmap is the ME that depicts the average gene expression throughout the module, and it demonstrates that the genes in this module are mostly upregulated in the lesioned hippocampus of animals with seizures. b) Network plot using VisAnt that shows the top three hundred connections within a module, where connection strength is calculated by TO [26]. The central positions of several genes are illustrated by this plot, including *Nfe2l2* and *Rdx*. c) Barplot showing fold changes in expression for six genes within the yellow module using qRT-PCR. The y-axis represents the fold changes between the right (lesioned; red) and left (non-lesioned; blue) hemispheres in epileptic and non-epileptic animals separately. Fold changes from the microarray data are shown for comparison. All genes show significantly higher levels of expression in the lesioned hemisphere of animals with seizures, while there was no significant change in animals without seizures (p<0.05). β-actin was used as a loading control for qRT-PCR. Error bars represent the standard deviation.

To examine the gene expression changes related to the epileptogenic region, we investigated the yellow module because its ME had the greatest correlation with the epileptogenic region. The yellow module contained 254 co-expressed genes that were specifically changed in the lesioned hippocampus of animals with seizures (Figure 5a). GO analysis demonstrated that these genes were involved in axon ensheathment (p = 2.11e-10) and glial cell differentiation (p = 1.08e-3) (Table S8). Using this module, we investigated the central genes and processes that were associated with epileptogenicity.

By determining the network position (*k*_ME_) of each of these genes, we were able to identify the central or hub within the module, and we found that the most highly connected gene was *Nfe2l2* (*k*_ME_ = 0.97), which remarkably, was previously found to be differentially expressed between the lesioned and non-lesioned hippocampi in animals with seizures. This gene and the other hubs within this module are illustrated in the network plot (Figure 5b). *Nfe2l2* is an important transcription factor involved in orchestrating the cellular response to oxidative stress [27]. Two of the known transcriptional targets of *Nfe2l2*, *Hmox1* and *Mgst1*, are also upregulated in this module and highly connected to *Nfe2l2*. In addition, proteins with glutathione S-tranferase activity are over-represented (p = 3.9e-2), which represent another aspect of the anti-oxidant system that is regulated by *Nfe2l2* [27], [28]. Therefore, several genes involved in the cellular response to oxidative stress are specifically upregulated within the epileptogenic region of animals with seizures. This unbiased, genome wide systems level analysis suggests that these hub genes play a central role in this process.

To confirm the expression changes that were observed in the yellow module, we used qRT-PCR on independent epileptic and non-epileptic samples. We validated several genes that were highly connected within yellow module, including *Nfe2l2* (*k*_ME_ = 0.97), *Hmox1* (*k*_ME_ = 0.88), *Mag* (*k*_ME_ = 0.95), *Klk6* (*k*_ME_ = 0.93), *Apod* (*k*_ME_ = 0.94), and *Rdx* (*k*_ME_ = 0.96) (Figure 5c). For each gene, we compared the expression in the lesioned hemisphere to expression in the non-lesioned hemisphere. In the animals with seizures, we observed significantly higher expression for each of these genes within the lesioned hemisphere (p<0.05). However, there was no significant change in the expression of any of these genes within the lesioned hemisphere of animals without seizures. Therefore, these data demonstrate that genes in the yellow module are consistently upregulated within the epileptogenic region, as hypothesized based on our bio-informatic analyses.

**Figure 6:**
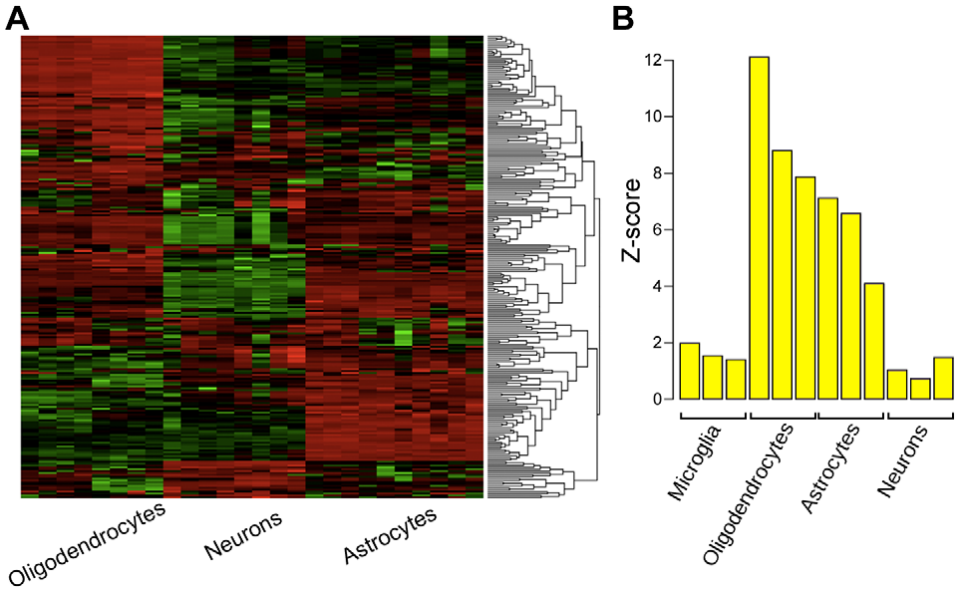
Co-expression module up-regulated within epileptogenic region contains genes expressed in glial cells. Using two resources of cellular specific expression, we examined whether the yellow module was enriched in genes that are expressed within glial cell types. a) Heatmap of genes in the yellow module using data extracted from a database of gene expression in oligodendrocytes, neurons, and astrocytes [20]. Samples are denoted across the columns and each row represents the scaled expression of a gene, where red and green signify higher and lower expression, respectively. The dendrogram was constructed by clustering the genes based on their Euclidean distance. b) Barplot showing the comparison of the yellow module to a resource that associates genes with modules using a connectivity value (k_ME_), where some of the modules have been shown to represent specific cell types [17]. For each module, we compared the average connectivity within the cell type specific modules of all genes in the yellow module to the average connectivity in these modules of a random group of genes. We expressed this comparison as a Z-score (shown in the barplot). There was significantly higher connectivity found in the modules that corresponded to astrocytes and oligodendrocytes than what would be expected by chance (p<1e-33 & p<1e-10).

Because several of the highly connected genes within the yellow module are expressed in glial cell sub-types, we hypothesized that this module was related to glial rather than neuronal changes within hippocampus. To move from whole tissue to cell type level analysis, we compared the composition of the yellow module to two previously published resources that explored gene expression within specific cell types. The first resource was a database that examined gene expression within the three major cell types in the brain, including astrocytes, oligodendrocytes, and neurons [20]. We located all of the genes in the yellow module in this database of gene expression and examined their expression by clustering the genes based on their Euclidean distances. Notably, we found that genes in the yellow module were consistently expressed in either oligodendrocytes or astrocytes and that very few of the genes were highly expressed in neurons (Figure 6a). To provide additional support for this analysis, we used the original human data set that provided the proof of principle demonstration that individual cell type analysis is feasible using WGCNA in whole brain tissue [17]. In this analysis, the authors annotated each gene by calculating its relationship, called *k*_ME_, with each module representing a cell type. For example, a gene with a greater *k*_ME_ with the neuronal module would be considered a neuronal gene, whereas a gene with a greater *k*_ME_ in the oligodendrocyte module would be considered an oligodendrocyte gene. We calculated the average *k*_ME_ for all genes in the yellow module within all of the cell type specific modules identified in Oldham et al., (2008), and we compared these values to random groups of genes and summarized the average *k*_ME_ values as Z-scores (Figure 6b). Consistent with the mouse data, we found that the genes within the yellow module had significantly higher connectivity with modules representing oligodendrocytes and astrocytes than by chance alone (p<1e-33 & p<1e-10).

These analyses indicate that this module largely corresponds to protection against oxidative stress in oligodendrocytes and astrocytes. The upregulation of genes involved in the anti-oxidant response suggests that there is ongoing oxidative stress in the lesioned hippocampus of animals with seizures, but not in animals that did not develop seizures. Therefore, these data represent the pathological processes that are associated with epileptogenicity and implicate glial dysfunction and response to oxidative stress as key components of this process.

### Chronic seizures cause dysregulation of synaptic vesicle trafficking within neurons

**Figure 7:**
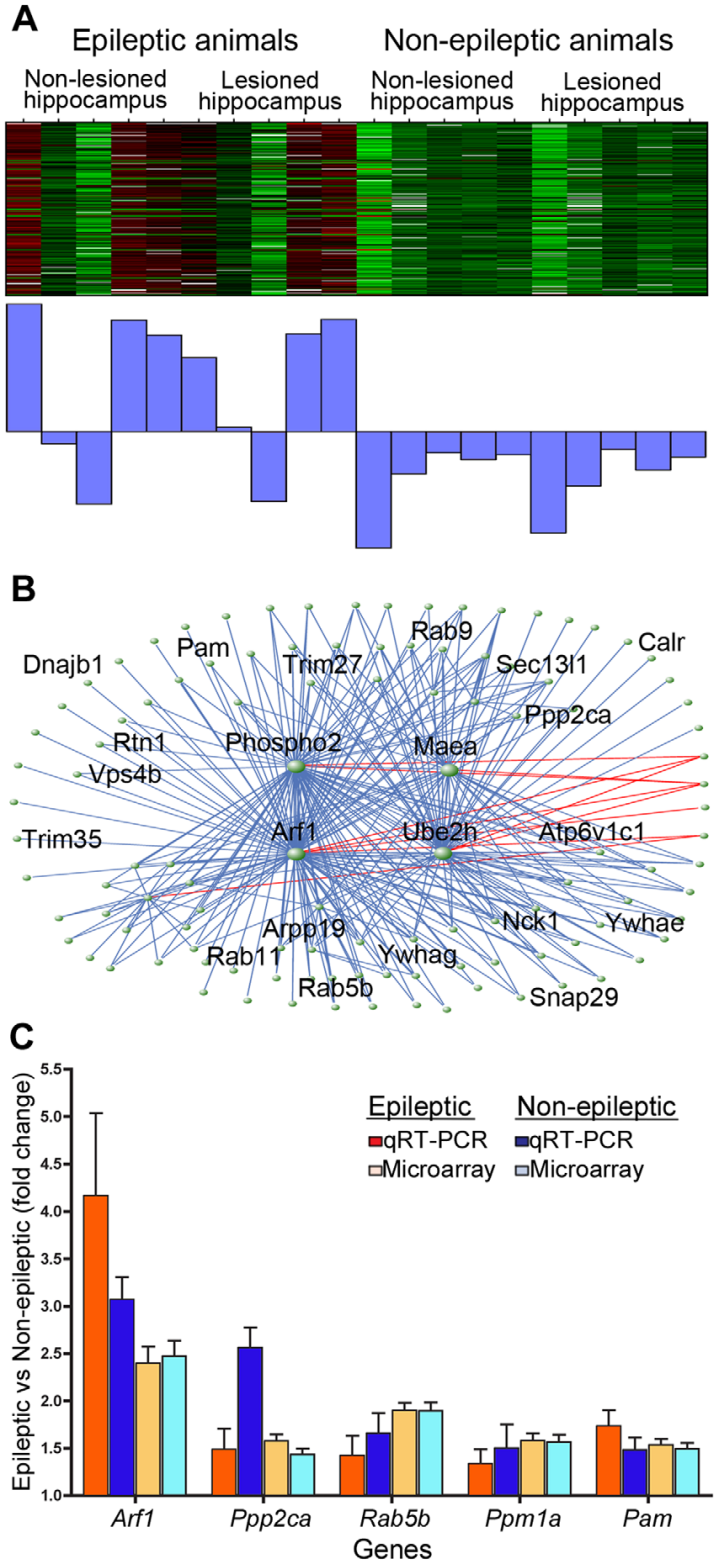
Co-expression module corresponding to effects of seizure suggests synaptic changes. The blue module contains genes that were upregulated in both hippocampi of animals with seizures. a) Heatmap of gene expression within this module, where samples are denoted across the columns and the genes are displayed in the rows. The barplot displays the ME that summarizes gene expression within the module. Although there is biological variability between the epileptic animals, these genes were mostly upregulated due to seizures regardless of lesion. b) In order to identify and highlight the most highly connected genes within the network, we plotted the top three hundred connections based on TO. The central position of *Arf1* within this module is clear, as well as three other genes *Phospho1*, *Maea*, and *Ube2h*. c) Barplot summarizing the fold changes of hubs within the blue module due to seizures in the lesioned (orange) and non-lesioned (blue) hemispheres, as measured by qRT-PCR. Fold changes calculated from the original microarray data are also shown for comparison. Each gene shows significantly higher levels of expression in animals with seizures than animals without seizures, regardless of hemisphere (p<0.05). β-actin was used as a loading control for qRT-PCR. Error bars represent the standard deviation.

We identified ten modules whose MEs were correlated with the effect of chronic seizures on the brain. Here, we focused on the blue module because it was the most highly correlated with the effects of seizures, although the ME demonstrates substantial biological variability between animals (Figure 7a). GO analysis demonstrated that the blue seizure-related module was highly enriched in genes involved in protein transport (p = 4.35e-7) and synaptic transmission (p = 0.014) (Table S9). These data are consistent with the differential expression analysis that suggested altered neurotransmission and show that the expression changes in genes involved in transport are co-regulated in the brain with chronic seizures. Within the blue module, *Arf1* is the most highly connected gene in the module (Figure 7b), and it is upregulated to a similar degree in both the lesioned and non-lesioned hippocampi. *Arf1* plays a role in vesicular trafficking and is specifically involved in synaptic vesicle biogenesis [29]. Previously, we observed that differentially expressed genes due to chronic seizures were involved in synaptic transmission (Table 4), and these data suggest that the blue module likely reflects alterations in synaptic vesicle trafficking.

**Table 4:** Hub genes within blue module differentially expressed due to seizures in both hippocampi.

Symbol	Name	*k*_ME_	Lesioned	Non-lesioned
Arf1	ADP-ribosylation factor 1	0.978	0.0076	0.0091
Rab5b	Rab5b, member ras oncogene family	0.973	5.2e-5	4.9e-5
Phospho2	Phosphatase, orphan 2	0.973	0.035	0.016
Ppp2ca	Protein phosphatase 2a, catalytic subunit, alpha	0.973	0.015	0.006
Zfp207	Zinc finger protein 207	0.972	1.4e-4	4.0e-4
Copa	Coatomer protein complex subunit alpha	0.972	0.0013	8.4e-4
Maea	Macrophage erythroblast attacher	0.972	0.018	0.062
Sumf1	Sulfatase modifying factor 1	0.971	0.011	0.0094
Ube2d3	Ubiquitin conjugating enzyme E2D3	0.970	0.022	0.014
Hnrnpc	Heterogeneous nuclear ribonucleoprotein C	0.970	4.9e-4	5.4e-4

The top ten most highly connected genes within the blue module and the significance (p-values) of their expression changes due to seizures in the lesioned and non-lesioned hemispheres. Genes within the table are ranked based on their k_ME_ within the blue module. For each gene, the last column shows the effect of seizures, which is the comparison between the animals with seizures and the animals without seizures. This comparison was performed separately in the lesioned and non-lesioned hippocampi, and the results of both analyses are shown in the table. All of these genes show similar trends towards differential expression in both the lesioned and non-lesioned hippocampi, demonstrating that their expression changes are likely caused by presence of seizures and are unrelated to the lesion.

We then confirmed the up-regulation of several hub genes within the blue co-expression due to the effect of chronic seizures. These genes included *Arf1* (*k*_ME_ = 0.97), *Ppp2ca* (*k*_ME_ = 0.97), *Rab5b* (*k*_ME_ = 0.97), *Ppm1a* (*k*_ME_ = 0.92), and *Pam* (*k*_ME_ = 0.92). We compared the expression of each of these genes between animals with seizures and animals without seizures. Expression in the lesioned hemisphere and non-lesioned hemisphere was compared independently, and we found that each gene was significantly upregulated due to the effect of chronic seizures in both hemispheres (Figure 7c). These data confirm the increase in expression of these genes observed with the microarrays.

**Figure 8:**
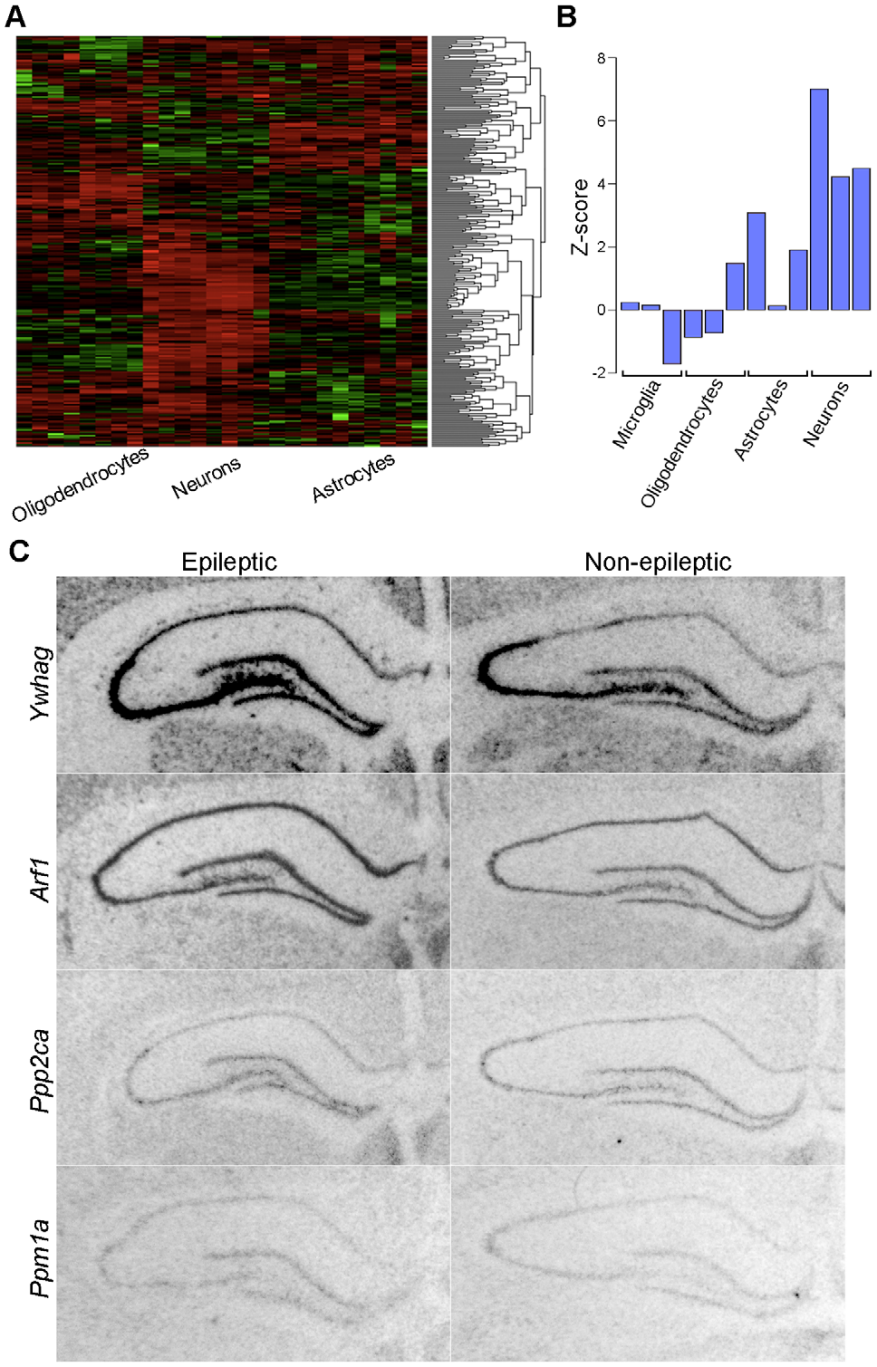
Blue module related to chronic seizures contains genes expressed in neurons. Due to the enrichment in synaptic functional categories of the yellow module, we examined the genes within the yellow module to determine whether they were enriched in neurons, using the same methods described for the yellow module (above). a) The expression values from the database of cellular gene expression [20] for the genes within this module were clustered based on Euclidean distance and displayed in the heatmap. The samples are denoted in the columns and the rows corresponded to the scaled expression of each gene, where red and green represent higher and lower expression, respectively. This heatmap shows that a majority of these genes are enriched in neurons, suggesting that neuronal gene expression is altered by seizures. b) We then used a second dataset a cell specific gene expression to examine the module from another perspective [17]. For each cell type specific module, we compared the average connectivity of the genes within the blue module to a random group of genes and expressed this comparison as a Z-score, which is shown in the barplot. We found that the blue module had over-represented connectivity with the neuronal modules, especially within the cortical neuron module (p<1e-10). c) To examine the expression of the genes within the seizure module directly, we used *in situ* hybridization. We examined the expression of *Ywhag* (*k*_ME_ = 0.92), *Arf1* (*k*_ME_ = 0.98), *Ppm1a* (*k*_ME_ = 0.97), and *Ppp2ca* (*k*_ME_ = 0.92) in the non-lesioned hemisphere of animals with (left) and without epilepsy (right). For each of these genes, there was little background signal, as examined using sense probes for each gene (Figure S2). Each gene was expressed within the neuronal layer of the dentate gyrus in animals with epilepsy, which is consistent with our hypothesis. Scale bar = 500 µm.

We then tested whether the genes that were upregulated due to seizures represented neuronal gene expression because many were involved in synaptic transmission. Using the same methods that were described for the yellow module above, we examined the cell types represented by the blue module. We identified all of the genes within the blue module in the database of gene expression in brain cell types [20], and we clustered these gene expression data based on the Euclidean distance between genes. This analysis revealed that the majority of the genes were highly expressed in neurons, while they were not consistently expressed in glial cell types (Figure 8a). To confirm these results, we examined the connectivity of the genes within the blue module to the cell type specific modules identified in human brain [17]. We observed significantly higher connectivity of the genes within the blue module to the module that represents neurons than by chance alone (Figure 8b; p<1e-10). These data extend the standard analysis of differential expression to demonstrate that the effects of chronic seizures cause dysregulation of synaptic vesicle trafficking within neurons.

To validate our hypothesis that the genes upregulated by seizures would increase their expression specifically within neurons, we used *in situ* hybridization to localize their expression within the hippocampus. We found that *Arf1* (*k*_ME_ = 0.98), Ywhag (*k*_ME_ = 0.92), Ppm1a (*k*_ME_ = 0.97), and *Ppp2ca* (*k*_ME_ = 0.92) all showed moderate to high expression within the granule cell layer of the dentate gyrus in both epileptic and non-epileptic animals (Figure 8c). Therefore, these data validate our hypothesis that the blue co-expression module represents the neuronal response to chronic seizures in the brain.

### Mechanisms of neuronal response to seizures

**Figure 9:**
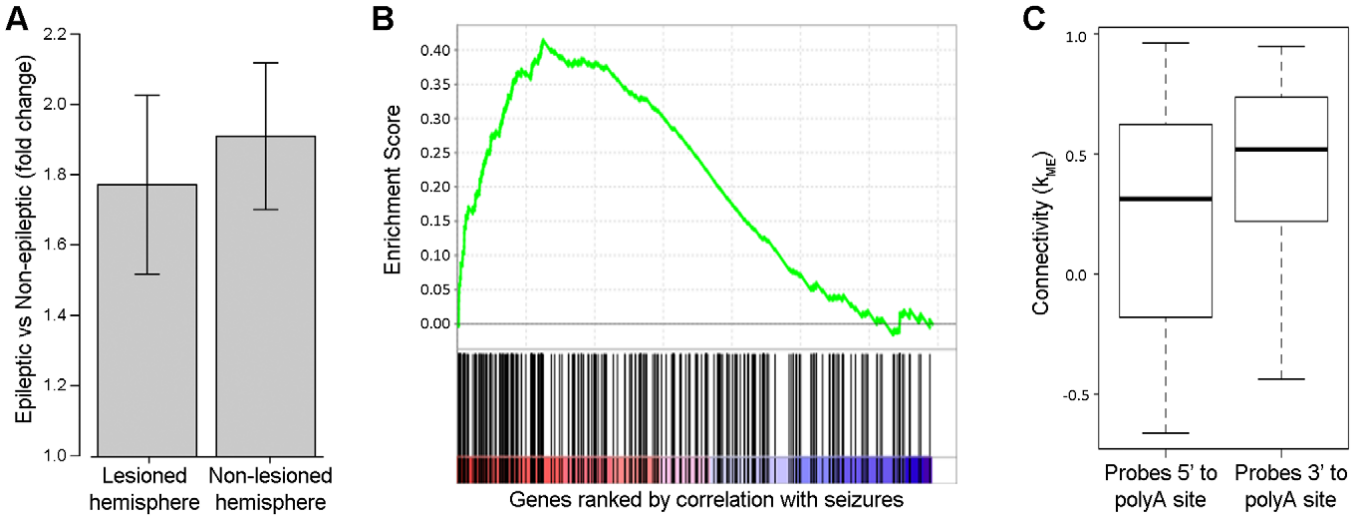
Involvement of HuD in regulating expression of genes within the yellow module. We examined the expression and role of HuD in modulating the response of neurons to chronic seizures. a) Barplot summarizing the fold changes of HuD within the blue module due to seizures in the right (lesioned) and left (non-lesioned) hemispheres separately. HuD shows significantly higher levels of expression in animals with seizures than animals without seizures, regardless of hemisphere (p<0.05). β-actin was used as a loading control for qRT-PCR. Error bars represent the standard deviation. b) Enrichment plot of HuD target genes showing their concordance with chronic seizures. The x-axis represents the list of genes that is ranked by their correlation with the seizure phenotype, where the red area denotes positive correlation with seizures and the blue area denotes negative correlation with seizures. The black lines on the x-axis show the location of the HuD target genes within the list of genes ranked by their correlation with seizures. The y-axis represents the enrichment score of HuD target genes within the ranked list of genes. This plot demonstrates that HuD targets show a significant correspondence with the effect due to chronic seizures (p<0.01). c) Boxplot showing the connectivity for probes interrogating regions 5′ to known polyadenylation sites and 3′ to known polyadenylation sites. The y-axis shows the distribution of connectivity (*k*_ME_) for the different groups of probes, where the thick black line represents the mean, the box represents one standard deviation, and the dotted lines represent three standard deviations. Probes that target regions that are 3′ to known polyadenylation sites demonstrate significantly higher connectivity than other probes that target regions that are 5′ to known polyadenylation sites (p<0.001).

The transcriptional changes due to seizures that are represented by the blue module may be caused by differences in transcriptional activation or changes in RNA processing. Since the RNA binding protein HuD has previously been suggested to contribute to the neuronal response to seizures [30], we investigated whether there was an over-representation of known targets of HuD [31]. Strikingly, among the genes that had high connectivity within the blue module (*k*_ME_>0.70), there was a highly significant over-representation of HuD targets (p = 2.12e-18), suggesting that HuD could play a role in regulating the transcripts within this module. To confirm that the expression of HuD was increased due to chronic seizures in this animal model, we performed qRT-PCR and found that HuD was significantly upregulated in both hemispheres of animals with seizures when compared to animals without seizures (Figure 9a).

We then hypothesized that targets of HuD would be upregulated due to seizures because HuD is known to bind to the 3′ UTR of its target genes and stabilize those transcripts [32], [33]. Therefore, we used Gene Set Enrichment Analysis [34] to determine whether previously identified targets of HuD [31] were up-regulated due to seizures. We found that this group of genes significantly corresponded to the effect of seizures (Figure 9b; p<0.01). These data demonstrate that the increase in HuD expression is accompanied by an increase in the functional consequences of the molecular functions of HuD.

Another known molecular function of HuD is the ability to interact with polyadenylation machinery and bias this process towards longer transcripts [35]. Therefore, we carefully examined the specific targets of all of the probes that interrogate HuD target genes. We found that probes targeting regions 3′ to known polyadenylation sites in HuD target genes were significantly more highly connected within the blue module than probes targeting regions 5′ to known polyadenylation sites in HuD target genes (p<0.001; Figure 9c). These data suggest that the up-regulation of HuD expression acts to bias polyadenylation site usage within its target genes. In addition, the high connectivity of target genes demonstrating altered polyadenylation provides a mechanism by which this HuD-mediated process likely contributes to the underlying regulation of the blue module.

### Epilepsy-specific changes in the synaptic vesicle trafficking module

**Figure 10:**
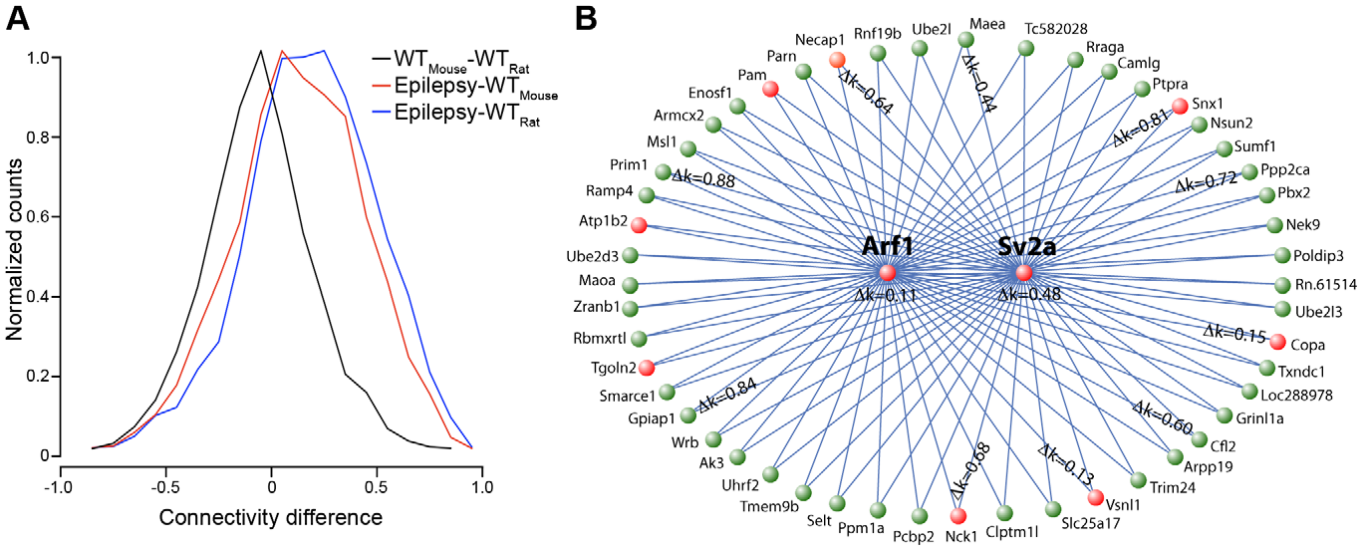
Genes involved in synaptic vesicle trafficking become more highly connected in epilepsy and are possible drug targets. We examined whether the seizure network was conserved across other datasets, using two published gene expression datasets. a) Histogram of differences in connectivity within the synaptic vesicle trafficking module between different gene expression studies, including our study of epilepsy, a study of expression in specific neuronal sub-types from mice (WT_Mouse_) [36], and a study of expression within the non-epileptic dentate gyrus from rats (WT_Rat_) [37]. The x-axis represents the connectivity difference is the difference in *k*_ME_ between the studies that are denoted on the legend. The y-axis represents the normalized number of genes that demonstrate the indicated connectivity difference. b) Network plot of the fifty closest genes to both *Arf1* and *Sv2a*, which were identified using the multi-point topological overlap measure [39]. Genes marked in red are known to be involved in vesicular transport or present in synaptic vesicles based on annotations from GO. For genes that had probes targeting the same exon in the epileptic and normal networks, we calculated the difference in k_ME_ between these networks and displayed these values on the network plot. Higher values demonstrate that the gene has become more important within the module, suggesting that it has gained a more substantial role in synaptic vesicle trafficking in neurons subject to seizure activity.

Because the blue module was enriched in neuronal genes and synaptic vesicle trafficking is common to all neurons, we hypothesized that this module should be present in other data sets; thus, finding such co-expression relationships independently would provide another level of validation of this module. We examined two different microarray datasets that examined gene expression in non-epileptic samples. The first dataset contained gene expression data from specific neuronal sub-types [36], and we used this dataset because the modules have previously been related to well-defined neuronal characteristics, permitting their annotation [18]. The second dataset contained gene expression data from the dentate gyri of animals involved in a learning study, and we used this dataset because the samples were comparable to our the tissue used in the current study [37]. We identified a module in each dataset that were enriched in genes involved in protein trafficking (p = 2.1e-6 & p = 2.5e-9) and had *Arf1* as a hub (*k*_ME_ = 0.91 & *k*_ME_ = 0.89). To determine whether these modules were truly comparable, we compared the top five hundred most highly connected genes in the epileptic seizure-related module and both of the non-epileptic modules, and we found a highly significant overlap in both comparisons (p<1.0e-10). These data both validate the co-expression relationships that we have identified using totally independent data sets and demonstrate that the functions of these modules are comparable. To determine whether connectivity within the seizure-related module was conserved, we compared the connectivity (*k*_ME_) of each gene in the seizure-related module to the conserved module of both non-epileptic datasets (Figure 10a). We observed a significant correlation between individual gene connectivities within each of these modules (p<1e-16), but there was a noticeable skewing of connectivity towards higher values in the epileptic module. These data suggest that some genes that had lower connectivity in normal animals have become more central in epilepsy. Interestingly, *Sv2a*, a synaptic vesicle glycoprotein and the molecular target of levetiracetam [38], has a much higher connectivity in the epileptic network (*k*_ME_ = 0.76), than in the non-epileptic modules (*k*_ME_ = 0.04 & *k*_ME_ = 0.52). These data suggest that *Sv2a* has gained a more important role in synaptic function in epilepsy than under normal conditions.

We hypothesized that other genes involved in synaptic vesicle trafficking that have high connectivity in the epilepsy network, but low connectivity in the normal network are possible drug targets for epilepsy. We further investigated this hypothesis using a measure called multi-point topological overlap [39], which identifies the nearest neighbors to a specific gene recursively based on their TO with the gene of interest. We identified the fifty nearest gene neighbors to *Sv2a* and *Arf1* using gene expression from epileptic animals (Figure 10b). Using GO, we found that this group of genes was enriched for genes localized to vesicles (p = 0.012). We then calculated the average difference in connectivity (*k*_ME_) in the epileptic and non-epileptic networks for each gene that had probes that targeted the same exon. We used this difference to prioritize the genes because genes that had large differences in connectivity between the networks were likely to be very important for the pathological processes that occur in chronic epilepsy (Table 5). For example, *Nck1* and *Necap1* are both known to be involved in vesicle trafficking [40], [41], and both are connected much more highly in the epileptic network than in the normal network. Therefore, these genes are anti-epileptic drug targets because we predict that they increase in importance under epileptic conditions and are part of the same pathway as the known drug target, *Sv2a*. However, there are other genes that are also highly dysregulated, but are not known to function in synaptic vesicle transport, such as *Ppp2ca*. These genes also warrant further study into both their possible role in synaptic vesicle trafficking, as well as the possibility that they are drug targets for epilepsy.

**Table 5:** Genes that displayed enhanced connectivity in epileptic vs. normal neurons.

Symbol	Gene title	Agilent	Sugino et al.	Burger et al.
Mrps25	Mitochondrial ribosomal protein S25	0.955	0.091	0.052
Prim1	DNA primase p49 subunit	0.944	0.043	0.088
Phospho2	Phosphatase, orphan 2	0.961	0.161	0.022
Hnrnpc	Heterogeneous nuclear ribonucleoprotein C	0.972	0.187	0.044
Dctn4	Dynactin 4	0.951	0.103	0.109
Pbx2	Pre B-cell leukemia transcription factor 2	0.938	0.076	0.114
Caprin1	GPI anchored membrane protein 1	0.950	0.032	0.190
App	Amyloid beta (A4) precursor protein	0.881	0.069	0.018
D3ucla1	DNA segment, Chr 3, UCLA 1	0.949	0.119	0.110
Pygb	Brain glycogen phosphorylase	0.892	0.091	0.034
Zadh2	Zinc binding alcohol dehydrogenase, 2	0.843	0.014	0.014
Socs7	Suppressor of cytokine signaling 7	0.905	0.100	0.085
Cdc5l	Cell division cycle 5-like	0.858	0.030	0.081
Parva	Parvin, alpha	0.802	0.003	0.002
3110048E14Rik	Riken cDNA 3110048E14 gene	0.829	0.033	0.051
Cdc42ep4	Cdc42 effector protein 4	0.914	0.050	0.207
Ngrn	Neugrin, neurite outgrowth associated	0.950	0.301	0.029
Nsg1	Neuron specific gene family member 1	0.901	0.136	0.106
Calm1	Calmodulin 1	0.906	0.003	0.255
Tmem93	Transmembrane protein 93	0.886	0.142	0.080

The top twenty genes that displayed the largest differences in connectivity between gene expression networks in epileptic and normal neurons. Three datasets were used to determine whether genes within the blue module displayed enhanced connectivity when compared to normal neuronal gene expression, including the Agilent data from the current study, a study of gene expression within normal neuronal sub-types [36], and a study of gene expression from the non-epileptic dentate gyrus [37]. Equivalent modules were identified in each dataset, and the connectivity (k_ME_) for genes that had probes targeting the same exon was compared in all modules. The values of connectivity are displayed in the table for the most differentially connected genes.

## Discussion

Here, we performed a genome-wide analysis of transcriptional changes in the KA lesion model of MTLE. By controlling for the effects of the initial neurotoxic hippocampal lesion, the study design allowed the identification of gene expression changes associated with epileptogenicity and homeostatic effects induced by seizures. Using differential expression analysis, we found that expression changes associated with epileptogenicity were important for neuronal survival and function, while chronic seizures caused changes in genes related to synaptic transmission. We examined these effects further using WGCNA and identified modules of highly co-expressed genes that corresponded to effects of epileptogenicity and seizures. The module that best corresponded to epileptogenicity suggested an increase in pathways that prevent damage due to oxidative stress in glial cells, extending the findings from the differential expression analysis and highlighting the role of glia in neuronal survival. We also examined another module that corresponded to the effects of chronic seizures on the brain, which may reflect homeostatic changes in response to seizure activity. This module contained genes involved in synaptic vesicle trafficking that were dysregulated in neurons from epileptic tissue. Notably, one hub within this module was the molecular target of levetiracetam, *Sv2a* [38], which suggests that pathways implicated by this module can modulate seizure activity and that other genes within this module are potential anti-epileptic targets.

One limitation of this study is that the animals in the gene expression studies were classified by observing convulsive seizures on video monitoring, but electrophysiological recordings were not conducted on the same group of animals. Thus, it is possible that animals in the non-epileptic gene expression group may have had non-convulsive seizures that could not be observed by video monitoring. This leads to the possibility that our control group may have been contaminated with animals that display some degree of electrophysiological seizure activity. In light of this possibility, we used WGCNA to obtain an unbiased perspective of the gene expression patterns present in all samples, and we found that none of the individual non-epileptic animals shared expression patterns with the epileptic animals. Therefore, it does not appear that gene expression in our control group was substantially affected by non-convulsive seizure activity. Alternatively, the expression changes observed in the epileptic animals may represent neuronal responses to chronic, recurrent convulsive seizures that were not engaged by non-convulsive seizures. Further studies with detailed electrophysiological characterization will be required to definitively associate these molecular and physiological changes.

Comparison of our data to previous studies was useful in contextualizing our results in terms of previously observed effects in animal models of MTLE. One major review of gene expression studies in epilepsy identified fifty-one consistently differentially expressed genes [42], and another large study of gene expression changes in kindled animals found 180 differentially expressed genes [12]. Expression changes reported by Lukasiuk and Pitkanen, (2004) overlapped with the differentially expressed genes due to the lesion in animals with seizures (n = 12 genes), but there was no overlap with either the epileptogenicity candidate genes or the epileptogenicity module. These data suggest that lesion-induced gene expression changes may have confounded the previous studies in this review. Genes found by Gorter et al., (2006) also had the largest overlap with differentially expressed genes due to the lesion in animals with seizures (n = 44 genes), but a subset of these genes overlapped with both the epileptogenicity candidate genes (n = 5 genes; *Bdnf*, *Fos*, *Grm4*, *Ptgds*, and *Unc13c*) and the epileptogenicity module (n = 8 genes; *Anxa1*, *Dbi*, *Fos*, *Ftl1*, *Hmox1*, *Mgst1*, *Prkcd*, and *Sdc4*). These data demonstrate that there are consistently differentially expressed genes due to epileptogenicity, which validates several of the changes observed in this study.

The first major goal of this study was to identify gene expression changes associated with the ability of the epileptogenic region to initiate spontaneous seizures or epileptogenicity. Using differential expression analysis, we found that lesion-induced changes were present in animals with and without epilepsy but that these changes were more dramatic in animals with seizures. Although we initially found that the lesions were grossly similar, these data suggest that the lesions were distinct molecularly. After correcting for lesion-induced changes, we identified forty candidate genes associated with epileptogenicity that were enriched for genes involved in neuronal survival. Interestingly, one candidate gene that was significantly upregulated in the epileptogenic region was *Fos*, which was also differentially expressed in Gorter et al., (2006). *Fos* is a transcription factor that immediately increases expression in response to seizure activity [43], and animals with a deletion of *Fos* demonstrated increased severity of KA-induced seizures and increased hippocampal cell death [44]. In addition, *Fos* has been shown to upregulate neuronal protective factors such as *Bdnf* [45], which is another epileptogenicity candidate gene in our analysis. In contrast to *Fos*, however, *Bdnf* was down-regulated in the epileptogenic region. This decrease in *Bdnf* expression has been described in other models of chronic epilepsy [46]. In addition, other neuropeptides, *Tac2* and *Cart*, were among the down-regulated epileptogenicity candidate genes, and these genes have roles in regulating both neuronal activity and survival [47], [48]. These data suggest that cells within the epileptogenic region are exposed to stressful conditions without a concomitant upregulation in factors that lead to survival. Therefore, we hypothesize that ongoing neuronal injury or death is associated with epileptogenicity.

We then used WGCNA to examine these important genes and processes associated with epileptogenicity from a systems perspective. The ME for the yellow co-expression module had the greatest correlation with epileptogenicity, although there was one animal without seizures that displayed up-regulation of these genes, which suggests that this animal may have had seizure activity without observable epilepsy. In addition, one of the epileptogenicity candidate genes, *Fos*, was highly connected within this module (*k*_ME_ = 0.85), providing further validation of these genes using another analytic method. The most highly connected gene within this module was the transcription factor *Nfe2l2* that is involved in the coordination of multiple different pathways that are protective against oxidative stress [27]. In addition, we found that the genes within the yellow module were mostly expressed in glia. Therefore, we hypothesized that this module may be related to epileptogenicity in a direct manner by altering the cellular composition of the lesioned area or in an indirect manner by signaling chronic oxidative stress.

The differential expression and network analyses make specific predictions about the processes and cell types that are altered in the epileptogenic region, which may provide insight into the mechanisms underlying epileptogenicity. It has been shown that there is increased glial differentiation from progenitor cells within the subgranular zone in KA-treated animals with spontaneous seizures [49]. This difference in cellular differentiation may be caused by changes in the cellular microenvironment [50], which we also observed through the differential expression of several neuropeptides. In addition, *Nfe2l2* knockout mice develop widespread astrogliosis and myelin degeneration, suggesting that *Nfe2l2* plays a role glial survival [28]. However, animals with a deletion of *Nfe2l2* are more susceptible to KA-induced seizures than wild-type animals [51]. These data suggest that production or protection of glial cell types is likely not involved in epileptogenicity, and therefore, the upregulation of this co-expression module is more likely secondary to chronic oxidative stress, which has been observed in this animal model [52]. Oxidative stress could account for the changes associated with neuronal injury identified in the differential expression analysis and has been shown to play a role in epileptogenicity. For example, mice that are heterozygous for a deletion in *Sod2* are more susceptible to KA-induced seizures and a subset of animals develops spontaneous seizures [53]. Oxidative stress is thought to increase epileptogenicity by inhibiting high affinity glutamate transporters in neurons and glia [53], [54], which are known to be sensitive to reactive oxygen species [55]. Therefore, these unbiased, genome-wide data lend support to the idea that the presence of oxidative stress, suggested by chronic upregulation of *Nfe2l2* within the lesioned hippocampus, may be a key biochemical feature responsible for epileptogenicity within that region.

Another goal of this study was to identify homeostatic responses to seizure activity that may be protective in epilepsy. Analysis of differential expression showed that the transcriptional response to seizures in the non-lesioned hippocampus was large and essentially equivalent to the response to seizures in the lesioned hippocampus. Although it intuitively makes sense that homeostatic responses to chronic, generalized seizures would affect both hippocampi equally, there are few studies that examine this phenomenon. For example, it has been shown that the expression of several neuropeptides is changed in non-lesioned hemisphere in the intrahippocampal KA animal model [56]. In addition, *Vglut1* can be upregulated in both hemispheres by a unilateral epileptogenic lesion [57], suggesting that seizure-induced synaptic plasticity is not restricted to the epileptogenic region. Consistent with these data, we observed that many genes involved in synaptic vesicle trafficking were differentially expressed due to seizures. Previous studies have shown that there is a redistribution of synaptic vesicles within the nerve terminal due to chronic seizures with vesicles shifted closer to the active zone [58]. In addition, the readily-releasable pool of glutamate has also been shown to be increased in the mossy fiber terminals of animals with epilepsy [59]. Therefore, these functional synaptic alterations that occur in response to epilepsy may be related to the gene expression changes that we observed.

To examine this phenomenon further, we investigated the blue module that was related to chronic seizures in both hippocampi, although there was substantial variability between animals, which could have been caused by a number of variables (ie. latency from last seizure, seizure frequency). This module was enriched in genes involved in synaptic transmission, and the most highly connected gene within this module was *Arf1*, which is a small GTPase that is involved in synaptic vesicle formation and trafficking [29], [60]. The main pathway represented by the blue module involves synaptic vesicle recycling, as *Arf1* is known to interact with the AP-3 complex and stimulate vesicle budding from the endosome [61]. Interestingly, it has been shown that neurons appear to have two simultaneous vesicle re-formation pathways [62], and *Arf1* is thought to be responsible for one of these endocytic processes [63]. Vesicles generated using *Arf1* and AP-3 have been shown to have unique properties and have altered release characteristics [64], [65]. By comparing out data with other datasets, we found that this hub gene (*Arf1*) and module were conserved but that the connectivity of many genes was increased in the epileptic module, suggesting that genes present in the blue module have gained new importance in the neurons of epileptic animals. These data lead to the hypothesis that granule cells in the dentate gyrus have altered synaptic vesicle retrieval because of an increased reliance on the *Arf1* and AP-3 dependent endocytic pathway, which could lead to changes in neurotransmission.

Several molecules and pathways may be involved in the underlying regulation of the genes within the blue module, but we identified one RNA binding protein, HuD, that may contribute significantly to these processes. However, we cannot discount other members of the Hu family. In fact, one previous study identified several hundred mRNAs that bind to HuR after pentylenetetrazol induced seizures [66]. We found a significant overlap between genes in the seizure module and these HuR targets (p = 2.11e-4), although this degree of overlap was than that of the HuD targets. The HuD protein has three RNA recognition domains that bind specifically to AU rich motifs that are present within the 3′ UTR many genes [32]. Functionally, over-expression of HuD within the hippocampus has also been shown to alter short and long-term plasticity, demonstrating that HuD can modulate neurotransmission [67], [68]. Although stabilization and increased translation of synaptic genes through HuD binding may cause these changes in synaptic function, alterations in polyadenylation site usage could also play a role. Many genes have multiple possible polyadenylation sites in the 3′ UTR, and transcripts terminated at different sites can have different functions [69]. For example, *Bdnf* has two known polyadenylation sites, leading to two populations of mRNA with different lengths [70]. Interestingly, only the longer transcript was found to be transported into the dendrites, and mice that lacked the longer isoform had dysmorphic dendritic spines and changes in synaptic physiology [70]. Therefore, alternative polyadenylation site usage of genes involved in synaptic vesicle trafficking due to seizures could alter their characteristics or function in several different ways, and these changes can modulate neuronal physiology and synaptic transmission.

Identification of gene expression changes caused by epilepsy and unassociated with epileptogenicity could provide important insights into natural neuronal mechanisms that alter neurotransmission, and these changes could represent a homeostatic, anti-epileptic response that limits seizure activity. Our data suggest that genes upregulated due to seizures may regulate neuronal excitability through effects on vesicular trafficking. Indeed, one of the hub genes within this module, *Sv2a*, is the target of the anti-epileptic drug levetiracetam and disruption of this gene causes seizures in mice [38], [71]. Although the function of *Sv2a* is not completely understood, it is localized to synaptic vesicles and influences neurotransmitter release by playing a role in vesicle priming [72], which are both related to the overall function associated with the module. These data suggest that modulation of pathways represented by this module can alter seizure activity and other genes within this module could be anti-epileptic drug targets. Few studies have attempted to identify drug targets from co-expression networks, but hub genes are thought to be the most efficient targets causing widespread network disruption [73], [74]. One study demonstrated that knockdown of hub genes in a specific type of brain cancer caused inhibition of cell division [75]. In addition, we have shown that the co-expression relationships that are defined by WGCNA reflect real, predictable changes that occur *in vivo* when hub genes are disrupted [18]. Therefore, hub genes in the epileptic network are likely to be the most efficient points of intervention within the module. Although current drug targets have been shown to be highly connected in protein interaction networks, they are also less likely to be essential for survival [76]. These data demonstrate a necessary balance between the importance of a gene in a pathological state and its importance for cellular survival. Therefore, genes that displayed lower connectivity in normal networks are likely to have more favorable toxicity profiles. Taken together, these data suggest that genes displaying increased connectivity in the epileptic network compared to the normal networks would be ideal drug targets. One such example is *Necap1*, a gene involved in synaptic vesicle endocytosis [41] that displays enhanced connectivity in the epileptic network. These data suggest that *Necap1* and other hub genes within the epileptic network are plausible therapeutic targets for inhibiting epileptiform activity in MTLE.

In summary, we have comprehensively examined gene expression changes in the KA model of MTLE. Our experimental design allowed us to identify genes associated with epileptogenicity, the lesion, and the effects of chronic seizures on the brain, which also permitted placing previous expression data in their appropriate context. These data show that many previously identified transcripts are related to confounding effects of the lesion. By performing complementary analyses of both differential expression and WGCNA at a genome-wide level, we were able to examine the effects of epilepsy from a systems level perspective. We found that glia and oxidative stress have prominent roles in contributing to epileptogenicity. Another key finding of this study was the identification and characterization of a homeostatic response to seizures, which involves changes in genes related to synaptic vesicle trafficking. These genes represent important alterations that affect the properties of synaptic transmission and include the anti-epileptic drug target *Sv2a*. Therefore, we hypothesize that these pathways associated with synaptic function modulate epileptiform activity and that genes identified in these analyses that are central to these processes represent plausible anti-epileptic drug targets (Table 5).

## Supporting Information

Figure S1 **Network construction and modular organization.** This dendrogram demonstrates a visual summary of the clustering of genes based on topological overlap. The network consists of approximately 11,000 genes that are assigned to 41 separate modules. A vertical line on the x-axis represents each gene, and the genes are grouped based on their topological overlap. The y-axis on the dendrogram represents the dissimilarity between neighboring genes on the dendrogram. Branches on the dendrogram represent co-expressed groups of genes (modules) that are isolated using an automatic module detection algorithm and assigned a color, which is shown on the horizontal bar below the dendrogram.
(TIF)

Figure S2 **In situ hybridizations of control sense probes.** Sense probes for *Arf1*, *Ppp2ca*, *Ywhag*, and *Ppm1a1* were used for *in situ* hybridization to show specificity of expression. *In situ* hybridizations using sense probes were carried out on adjacent sections to those that were used for antisense probes. Sense and antisense probes were hybridized to sections under the same conditions at the same time.
(TIF)

Table S1 **Primers used for quantitative RT-PCR.** This table shows the primers that were used for quantitative RT-PCR confirmation. The first column shows the gene symbol. The second column shows the genomic location that was targeted. The third column represents the forward primer in 5′→3′ direction. The fourth column represents the reverse primer in 5′→3′ direction.
(XLS)

Table S2 **Primer and probe sequences used for** ***in situ*** **hybridization.** This table shows the primer and probe sequences that were used for in situ hybridization. The first column shows the gene symbol. The second column shows the genomic location that was targeted. The third column shows the primers in 5′→3′ direction that were used to clone the probe sequences. The fourth column shows the entire probe sequence that was used for hybridization.
(XLS)

Table S3 **Summary of tissue samples used for microarrays.** This table summarizes the tissue samples that were used for gene expression studies. The first column shows the animal identifier. The second column denotes the results of the video monitoring. The third column shows the hemisphere that the sample was taken from. The fourth column shows the number of sections that were used for mRNA extraction. The fifth column shows thickness of the sections that were used. The sixth column represents the concentration of mRNA that was extracted. The seventh column shows the 260/280 ratio of the extracted mRNA. The eighth column shows the results from the Agilent Bioanalyzer.
(XLS)

Table S4 **Differentially expressed genes due to the lesion in animals without seizures.** This table summarizes the genes that are differentially expressed between the lesioned and non-lesioned hippocampi in animals without seizures. The gene symbols, gene names, and Refseq identifiers are shown in the first column. The probe on the Agilent platform and the fold change and differential expression p-value for that probe is shown in the fourth, fifth, and sixth columns. The probe on the Codelink platform and the fold change and differential expression p-value for that probe is shown in the seventh, eighth, and ninth columns.
(XLS)

Table S5 **Differentially expressed genes due to the lesion in animals with seizures.** This table summarizes the genes that are differentially expressed between the lesioned and non-lesioned hippocampi in animals with seizures. The gene symbols, gene names, and Refseq identifiers are shown in the first column. The probe on the Agilent platform and the fold change and differential expression p-value for that probe is shown in the fourth, fifth, and sixth columns. The probe on the Codelink platform and the fold change and differential expression p-value for that probe is shown in the seventh, eighth, and ninth columns.
(XLS)

Table S6 **Differentially expressed genes due to seizures in the non-lesioned hippocampus.** This table summarizes the genes that are differentially expressed between lesioned hippocampi in animals with and without seizures. The gene symbols, gene names, and Refseq identifiers are shown in the first column. The probe on the Agilent platform and the fold change and differential expression p-value for that probe is shown in the fourth, fifth, and sixth columns. The probe on the Codelink platform and the fold change and differential expression p-value for that probe is shown in the seventh, eighth, and ninth columns.
(XLS)

Table S7 **Differentially expressed genes due to seizures in the lesioned hippocampus.** This table summarizes the genes that are differentially expressed between the non-lesioned hippocampi in animals with and without seizures. The gene symbols, gene names, and Refseq identifiers are shown in the first column. The probe on the Agilent platform and the fold change and differential expression p-value for that probe is shown in the fourth, fifth, and sixth columns. The probe on the Codelink platform and the fold change and differential expression p-value for that probe is shown in the seventh, eighth, and ninth columns.
(XLS)

Table S8 **Functional annotation of the module associated with epileptogenicity.** Enriched GO categories for the genes present within the yellow module that was specific to the epileptogenic region. The first column shows whether the categories are associated with a biological process, cellular component, or molecular function. Specific, enriched functional categories are shown in the second column. The third and fourth columns show the number and percentage of genes in the module that are associated with the specific term. The fifth column shows enrichment p-value.
(XLS)

Table S9 **Functional annotation of the module associated with the effect of seizures.** Enriched GO categories for the genes present within the blue module that corresponded to both hippocampi that were subject to seizures. The first column shows whether the categories are associated with a biological process, cellular component, or molecular function. Specific, enriched functional categories are shown in the second column. The third and fourth columns show the number and percentage of genes in the module that are associated with the specific term. The fifth column shows enrichment p-value.
(XLS)
