# Supplementary figures and images for: A Systems Level, Functional Genomics Analysis of Chronic Epilepsy
Source: PLoS One. 2011 Jun 14;6(6):e20763. doi: 10.1371/journal.pone.0020763 (PMC3114768; doi:10.1371/journal.pone.0020763)

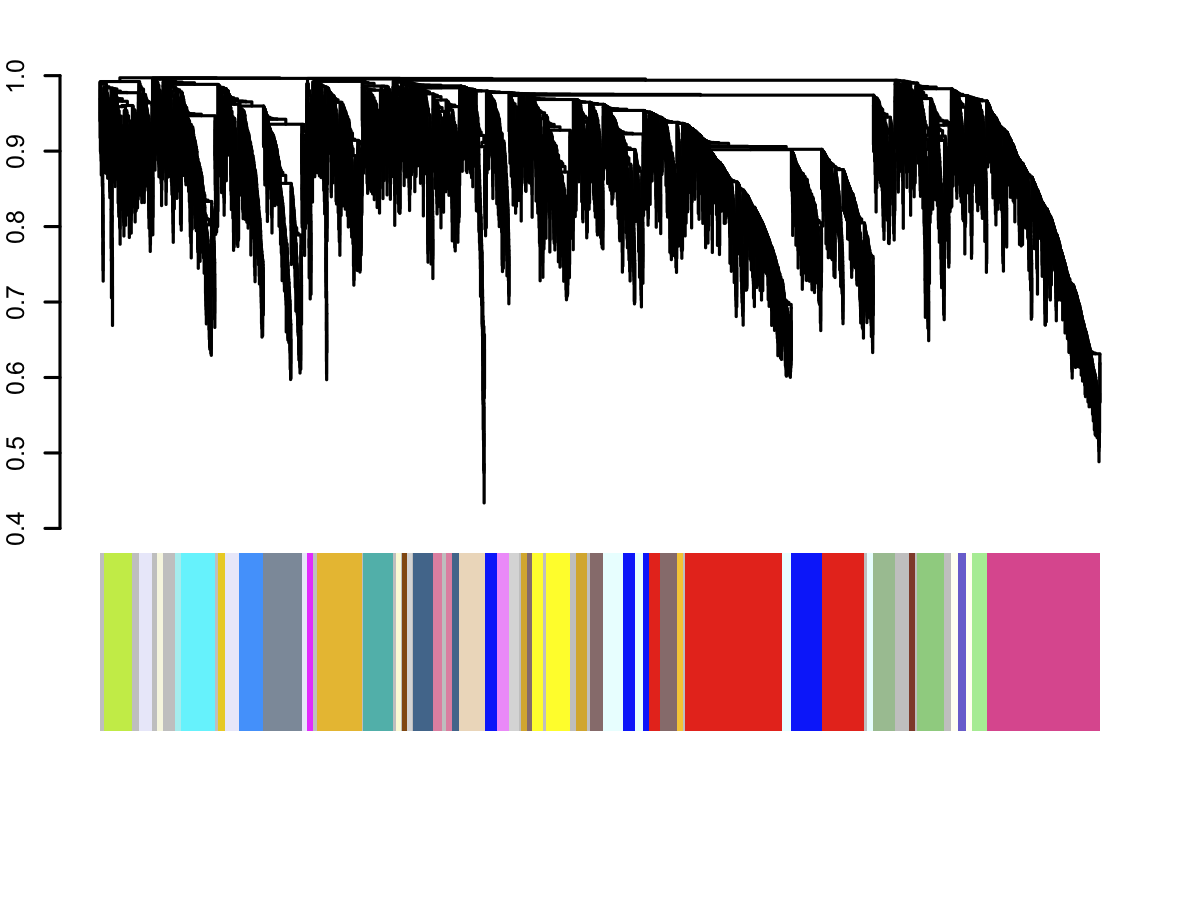

Supplement: Figure S1 — Network construction and modular organization. This dendrogram demonstrates a visual summary of the clustering of genes based on topological overlap. The network consists of approximately 11,000 genes that are assigned to 41 separate modules. A vertical line on the x-axis represents each gene, and the genes are grouped based on their topological overlap. The y-axis on the dendrogram represents the dissimilarity between neighboring genes on the dendrogram. Branches on the dendrogram represent co-expressed groups of genes (modules) that are isolated using an automatic module detection algorithm and assigned a color, which is shown on the horizontal bar below the dendrogram. (TIF) [file pone.0020763.s001.tif]

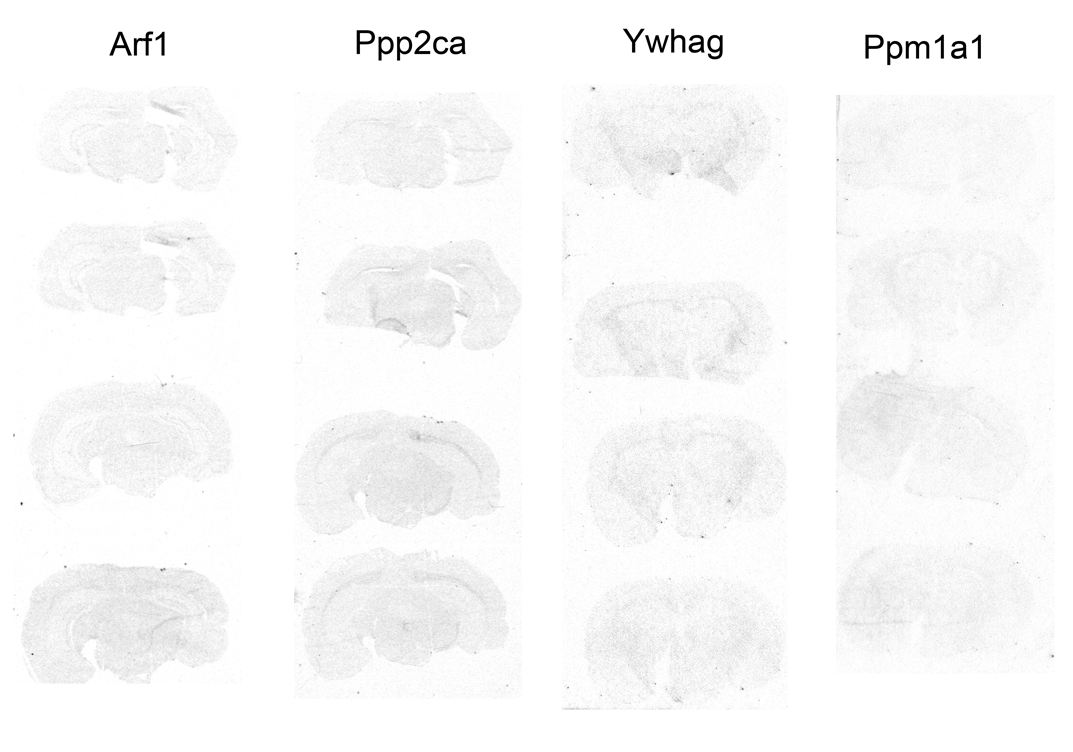

Supplement: Figure S2 — In situ hybridizations of control sense probes. Sense probes for Arf1, Ppp2ca, Ywhag, and Ppm1a1 were used for in situ hybridization to show specificity of expression. In situ hybridizations using sense probes were carried out on adjacent sections to those that were used for antisense probes. Sense and antisense probes were hybridized to sections under the same conditions at the same time. (TIF) [file pone.0020763.s002.tif]
